# Actin blobs prefigure dendrite branching sites

**DOI:** 10.1083/jcb.201711136

**Published:** 2018-10-01

**Authors:** Vanitha Nithianandam, Cheng-Ting Chien

**Affiliations:** 1 Molecular and Cell Biology, Taiwan International Graduate Program, Academia Sinica and Graduate Institute of Life Sciences, National Defense Medical Center, Taipei, Taiwan; 2 Institute of Molecular Biology, Academia Sinica, Taipei, Taiwan

## Abstract

The actin cytoskeleton provides structural stability and adaptability to the cell. Neuronal dendrites frequently undergo morphological changes by emanating, elongating, and withdrawing branches. However, the knowledge about actin dynamics in dendrites during these processes is limited. By performing in vivo imaging of F-actin markers, we found that F-actin was highly dynamic and heterogeneously distributed in dendritic shafts with enrichment at terminal dendrites. A dynamic F-actin population that we named actin blobs propagated bidirectionally at an average velocity of 1 µm/min. Interestingly, these actin blobs stalled at sites where new dendrites would branch out in minutes. Overstabilization of F-actin by the G15S mutant abolished actin blobs and dendrite branching. We identified the F-actin–severing protein Tsr/cofilin as a regulator of dynamic actin blobs and branching activity. Hence, actin blob localization at future branching sites represents a dendrite-branching mechanism to account for highly diversified dendritic morphology.

## Abstract (precis)

Nithianandam and Chien show via in vivo imaging that a dynamic population of F-actin termed actin blobs propagates bidirectionally in dendrites and stalls at future branching sites. The F-actin–severing protein Tsr/cofilin is a regulator of actin blob dynamics and dendrite branching.

## Introduction

The actin cytoskeleton, depending on cellular needs, shapes into various dimensions in a spatiotemporal manner. In neurons, actin is essential for morphological changes such as neurite formation and growth cone navigation. Initially, monomeric actin polymerizes to form F-actin that is further organized into higher-order dynamic assemblies like actin waves and trails (Allard and Mogilner, 2013; Roy, 2016; Inagaki and Katsuno, 2017). In cultured hippocampal neurons, actin waves propagate from the base to the tip of the neurite once every half hour at a velocity of 3 µm/min (Ruthel and Banker, 1999; Flynn et al., 2009). Actin waves could contribute to axonogenesis as the waves appear with higher frequencies in the early than later developmental stages in neurites that would become axons. Actin trails, propagated in a different dynamic manner, nucleate and elongate from stationary endosomes in the axonal shaft at a fast rate of 1 µm/s (Schuh, 2011; Ganguly et al., 2015). The putative function of actin trails is to deliver F-actin to presynaptic regions for synaptic membrane recycling. These actin assemblies depend on different actin regulatory proteins for their genesis and motility. For instance, the actin nucleator Arp2/3 and microtubules are required for the propagation of actin waves but dispensable for actin trail elongation (Ruthel and Banker, 1998; Ganguly et al., 2015; Katsuno et al., 2015). Hence, these dynamic F-actin populations with distinct characteristics contribute to different functions in the axon.

In addition to axons, neuronal dendrites also contain dynamic actin assemblies. In cultured hippocampal neurons, actin waves propagate from the base to dendritic ends with a putative role in dendrite growth (Ruthel and Banker, 1999). Distinct actin organizations such as longitudinal actin, actin patches, and rings are present in dendrites of cultured hippocampal neurons (Xu et al., 2013; D’Este et al., 2015; Bär et al., 2016). However, dynamic natures and functions of these actin organizations remain to be determined. In dendritic spines, actin organization varies in different spine compartments. While branched networks of F-actin are pronounced in the head, linear actin structures are evident in the neck (Korobova and Svitkina, 2010). Furthermore, actin dynamics also vary at different spine regions. A dynamic pool of F-actin produces expansive force at the spine tip, whereas a stable pool of F-actin at the spine base possibly contributes to stability (Honkura et al., 2008). Currently, while some actin assemblies in axons and dendritic spines have been known, the knowledge of actin organizations and dynamics in dendritic shafts is incomplete (Konietzny et al., 2017).

Genetic studies have shed light on the role of actin-binding proteins in regulating dendrite branching. Loss of Enabled (Ena), an actin-polymerizing factor, causes a reduction in dendrite branching, whereas the actin nucleator Spir is involved in dendrite patterning (Gao et al., 1999; Ferreira et al., 2014). The small GTPase proteins Rac1 and Cdc42 positively regulate dendrite branching, whereas RhoA inhibits the process (Lee et al., 2000, 2003; Ng et al., 2002; Scott et al., 2003). In hippocampal neurons, Cobl mediates F-actin nucleation before dendrite branching (Hou et al., 2015). Similarly, in *Drosophila melanogaster* class III dendritic arborization (da) neurons, local actin accumulation precedes the formation of spike-like terminals (Andersen et al., 2005). It is not clear whether local F-actin nucleation and accumulation are also applicable to other types of neurons. Fascin, an actin-bundling protein, localizes specifically to the terminal spikes of class III da neurons and is essential for spike formation. However, Fascin is dispensable for dendrite branching in class IV da neurons (Nagel et al., 2012). Considering the huge diversity of neurons in terms of dendritic morphology, these analyses only reveal a subset of actin-regulatory mechanisms in dendrites.

In this study, we examined actin dynamics in class IV da neurons that display the highest complexity among four classes of da neurons in *Drosophila* (Grueber et al., 2002). In addition to the uneven distribution of F-actin that reflects the dynamic nature of dendrites, we identified a population of F-actin assemblies we named “actin blobs.” We report the features of actin blobs in dendrites and, more importantly, their prelocalization to future branching sites. Localization of dynamic actin blobs at the branching sites represents a distinct mechanism to previously described F-actin nucleation at the branching sites. By a genetic screen for actin-regulatory factors, we identified Twinstar (Tsr), the *Drosophila* cofilin homologue (Gunsalus et al., 1995), to regulate the actin blob genesis in dendrites. Cofilin, an F-actin–severing protein, binds to F-actin and induces breakage of F-actin at the site between cofilin-bound and -unbound regions (Prochniewicz et al., 2005). The severed F-actin produces free barbed ends that can be depolymerized or further nucleate actin polymerization (Ichetovkin et al., 2002; Okreglak and Drubin, 2010). In dendritic spines, cofilin regulates both spine shrinkage and enlargement (Racz and Weinberg, 2006; Calabrese et al., 2014; Noguchi et al., 2016). However, how the loss of cofilin affects dendrite branching remained elusive. By studying *tsr* mutant defects, we propose that actin blob regulation by Tsr is important for dendrite branching. Further study of the actin variant G15S that stabilizes F-actin due to resistance to cofilin binding also confirms the importance of actin blob regulation in dendrite branching.

## Results

### Actin blobs, a population of dynamic F-actin assemblies in dendrites

**Figure 1.**
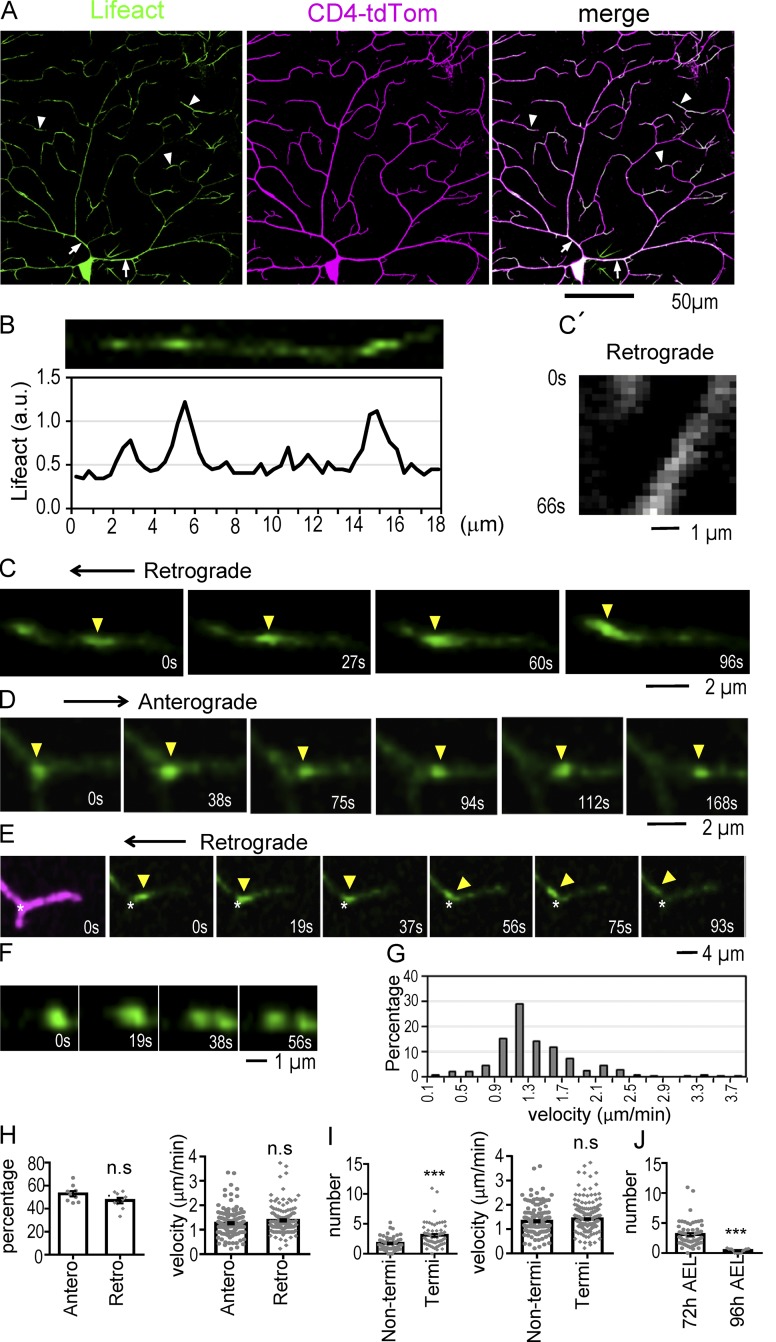
**Distribution and dynamics of actin blobs in dendrites. (A)** Distribution of LifeAct (green) expressed by *ppk-GAL4* in dendritic arbors of class IV da neurons marked by *ppk-CD4-tdTomato* (magenta). Arrows indicate high LifeAct signals in proximal dendrites, and arrowheads indicate these in terminal dendrites. **(B)** A terminal dendrite was straightened (top) to show uneven distribution of LifeAct signals. LifeAct intensities normalized to tdTomato intensities were shown along the shaft (bottom). x axis, μm; y axis, AU. **(C)** Time series images show actin blob propagation in the retrograde direction (see also Video 1). **(C′)** Kymograph shows changes of LifeAct intensities along the dendritic shaft (x axis) and time (0–66 s; y axis). **(D–F)** Time series images show anterograde movement of an actin blob (D; Video 2), the passage of an actin blob through a branching site indicated by asterisks (E; Video 3), and actin blob splitting (F; Video 4). **(G)** Bar graph shows percentages (y axis) of actin blobs versus velocities with a 0.2-µm/min increment (x axis). In total, 404 actin blobs in 164 dendrites of nine neurons in five experiments were recorded. **(H)** Bar graphs compare percentages (left; dot represents a neuron) and velocities (μm/min; right; dot represents a blob) of actin blobs between anterograde (Antero) and retrograde (Retro) propagation. In total, 152 actin blobs from nine neurons for retrograde and 160 actin blobs in nine neurons for anterograde were recorded. **(I)** Comparing actin blob numbers (in 10 µm; left; dot represents a dendrite segment) and velocities (μm/min; right) in nonterminal (Non-termi; 156 actin blobs from 50 dendrites) and terminal (Termi; 248 actin blobs from 69 dendrites) dendrites. **(J)** Comparing actin blob numbers in 10-µm terminal dendrites in early third (72 h AEL; replicate of terminal dendrites in I) and mid–third instar (96 h AEL; 15 dendrites from three neurons). Significance was determined using Student’s *t* test. ***, P < 0.001. Error bars represent SEM.

To delineate the elusive role of actin in dendritic shafts, we expressed LifeAct in class IV da neurons by *ppk-GAL4,* allowing us to visualize F-actin in vivo (Riedl et al., 2008). We observed that the F-actin was heterogeneously distributed in dendritic shafts with higher intensities in proximal and terminal dendrites (Fig. 1 A, arrows and arrowheads, respectively). These high-intensity F-actin signals were intermittently distributed along dendritic shafts, as shown by quantification of LifeAct intensities along a segment (Fig. 1 B).

To further characterize F-actin distribution in dendrites, we performed live imaging. Interestingly, high-intensity F-actin signals were dynamic in nature (Fig. 1, C–F; and Videos 1, 2, 3, and 4). Some F-actin aggregates appeared rounded, while some others were in elongated shapes. These dynamic clusters of F-actin propagating in dendrites termed actin blobs were present in all regions of the dendritic arbor. The size of these actin blobs ranged from 1 to 6 µm in length along the dendritic process, with an average size of 3.1 µm (refer to Table 1 for SD and sample numbers hereafter). Propagation of actin blobs was bidirectional in both retrograde (toward cell body) and anterograde (toward dendritic tip) directions (Fig. 1, C and D; and Videos 1 and 2). Within a 10-min imaging period, almost all dendrites (96.6%) had actin blobs, with an average of 2.5 actin blobs in 10 µm of length. We also observed actin blob turning at branch points, moving from the terminal dendrite to the mother dendrite (Fig. 1 E and Video 3). Splitting of actin blobs was detected frequently (Fig. 1 F and Video 4). In 10 min, 42.1% of dendrites had at least a splitting event. The two daughter actin blobs after splitting could move independently. The velocity of actin blob movement was quantified using kymographs (Fig. 1 C′). We found that the velocities of actin blobs varied, with the majority of them (>77%) moving at speeds between 0.9 and 1.9 µm/min (Fig. 1 G). The average velocity was 1.4 µm/min.

**Table 1.** Features of actin blobs

**Description**	**Average ± SD**^a^ **(sample number)**	
	**LifeAct**	**GMA**
Actin blob size (μm)	3.1 ± 1.1 (*n* = 68)	3.5 ± 1.6 (*n* = 32)
Dendrites with actin blobs (%)	96.6 (*n* = 119)	92.0 (*n* = 44)
Actin blob number (in 10-µm length)	2.5 ± 1.8 (*n* = 119)	1.8 ± 1.2 (*n* = 44)
Actin blob splitting event (% in 10-µm dendrite)	42.1 (*n* = 38)	28.6 (*n* = 35)
Actin blob velocity (μm/min)	1.4 ± 0.6 (*n* = 404)	2.1 ± 0.7 (*n* = 121)
Anterograde actin blob (%)	52.9 (*n* = 9)	50.7 (*n* = 7)
Retrograde actin blob (%)	47.1 (*n* = 9)	49.3 (*n* = 7)
Anterograde velocity (μm/min)	1.3 ± 0.5 (*n* = 160)	2.1 ± 0.8 (*n* = 63)
Retrograde velocity (μm/min)	1.4 ± 0.5 (*n* = 152)	2.0 ± 0.7 (*n* = 58)
Number in terminal dendrites (in 10 µm)	3.1 ± 1.9 (*n* = 69)	2.2 ± 1.3 (*n* = 28)
Number in nonterminal dendrites (in 10 µm)	1.8 ± 1.2 (*n* = 50)	1.1 ± 0.7 (*n* = 16)
Velocity in terminal dendrites (μm/min)	1.4 ± 0.5 (*n* = 248)	2.1 ± 0.7 (*n* = 88)
Velocity in nonterminal dendrites (μm/min)	1.3 ± 0.6 (*n* = 156)	2.0 ± 0.7 (*n* = 33)
Number in mid–third instar stage (in 10 µm)	0.4 ± 0.3 (*n* = 15)	0.8 ± 0.6 (*n* = 14)
Number in class III da neurons (in 10 µm)	0.6 ± 0.9 (*n* = 20)	ND^b^
Velocity in class III da neurons (μm/min)	1.8 ± 0.5 (*n* = 11)	ND

a When applicable.

b Not done.

We further examined any difference between anterograde and retrograde propagations. Almost equal percentages of anterograde (52.9%) and retrograde (47.1%) propagations were present with similar velocities (Fig. 1 H). In 10 µm of terminal dendrites, 3.1 actin blobs were detected, and the same length of nonterminal dendrites had 1.8 actin blobs. Thus, terminal dendrites that undergo frequent extension and retraction had a higher number of actin blobs, although both populations of actin blobs propagated at similar velocities (Fig. 1 I). In addition, actin blob propagation was developmentally regulated. Compared with the early third instar stage (72 h after egg laying; AEL), the number of actin blobs in terminal dendrites was drastically reduced in the mid–third instar stage (96 h AEL; Fig. 1 J). Therefore, we focused our study of actin blobs in dendrites in the early third instar stage.

We investigated whether actin blob movement depends on microtubules. In RNAi knockdown against *αTub84B*, deficiency in microtubules was revealed by the reduction in signal intensities of microtubule-associated Jupiter-Cherry as well as Futsch and α-tubulin immunostaining (Fig. S1, A–D). *αTub84B* knockdown also caused defects in dendrite morphogenesis (Fig. S1 A). However, the number of actin blobs per 10 µm dendrites and the velocity of propagation remained the same (Fig. S1 F; see Table S1 for SD and sample numbers). To further address the role of microtubules in regulating actin blobs, we overexpressed the microtubule-severing protein Katanin 60 (Kat60), which destabilizes microtubules and alters dendrite morphology (Mao et al., 2014). While we detected consistent results as reported, Kat60 overexpression failed to reduce the actin blob number with slightly increased velocity (Fig. S1, E and F). Put together, microtubules are largely dispensable to actin blob propagation in dendrites.

Dendrite branching remained normal with LifeAct expression (Fig. S2 A). To further validate the observed F-actin dynamics in dendrites, we employed an alternative F-actin probe GMA consisting of GFP fusion to the actin-binding domain of moesin (Edwards et al., 1997). Similar to LifeAct expression, the dendrite pattern was normal upon GMA expression (Fig. S2 A). GMA-labeled actin blobs had essentially the same properties such as distributions and velocities (Fig. S2, B–F; Video 5; and Tables 1 and 2), justifying that LifeAct is a suitable reporter probing dynamic F-actin in vivo.

**Table 2.** Actin blobs in dendrite branching, retraction, and extension

**Description**	**Value**^a^ **(sample number)**	
	**LifeAct**	**GMA**
New branches with actin blob prelocalization (%)	83.0 (*n* = 53)	87.5 (*n* = 24)
Local F-actin accumulation before branching (%)	11.3 (*n* = 53)	8.3 (*n* = 24)
No F-actin enrichment before branching (%)	5.7 (*n* = 53)	4.2 (*n* = 24)
Anterograde actin blob to branching site (%)	27.3 (*n* = 44)	
Retrograde actin blob to branching site (%)	54.5 (*n* = 44)	
Retro- and anterograde actin blobs to branching site (%)	18.2 (*n* = 44)	
Time of localization before dendrite branching (s)	114 (*n* = 41)	
Retracting dendrites with retrograde actin blobs (%)	33.3 (*n* = 21)	
Retracting dendrites with anterograde actin blobs (%)	9.5 (*n* = 21)	
Retracting dendrites with two actin blobs (%)	33.3 (*n* = 21)	
Retracting dendrites with no actin blobs (%)	23.8 (*n* = 21)	
Extending dendrite with retrograde actin blobs (%)	57.1 (*n* = 21)	
Extending dendrite with anterograde actin blobs (%)	9.5 (*n* = 21)	
Extending dendrite with two actin blobs (%)	19.1 (*n* = 21)	
Extending dendrite with no actin blobs (%)	14.3 (*n* = 21)	
Actin blob stalling followed by branching (%)	24 (*n* = 49)	
Actin blobs localizing at the branching site in class IV (%)	71 (*n* = 24)	
Actin blobs localizing at the branching site in class III (%)	70 (*n* = 10)	
Class III spike formation with actin blob prelocalization (%)	35 (*n* = 20)	

a Values correspond with LifeAct unless otherwise mentioned.

### Actin blob prelocalization at dendrite branching sites

**Figure 2.**
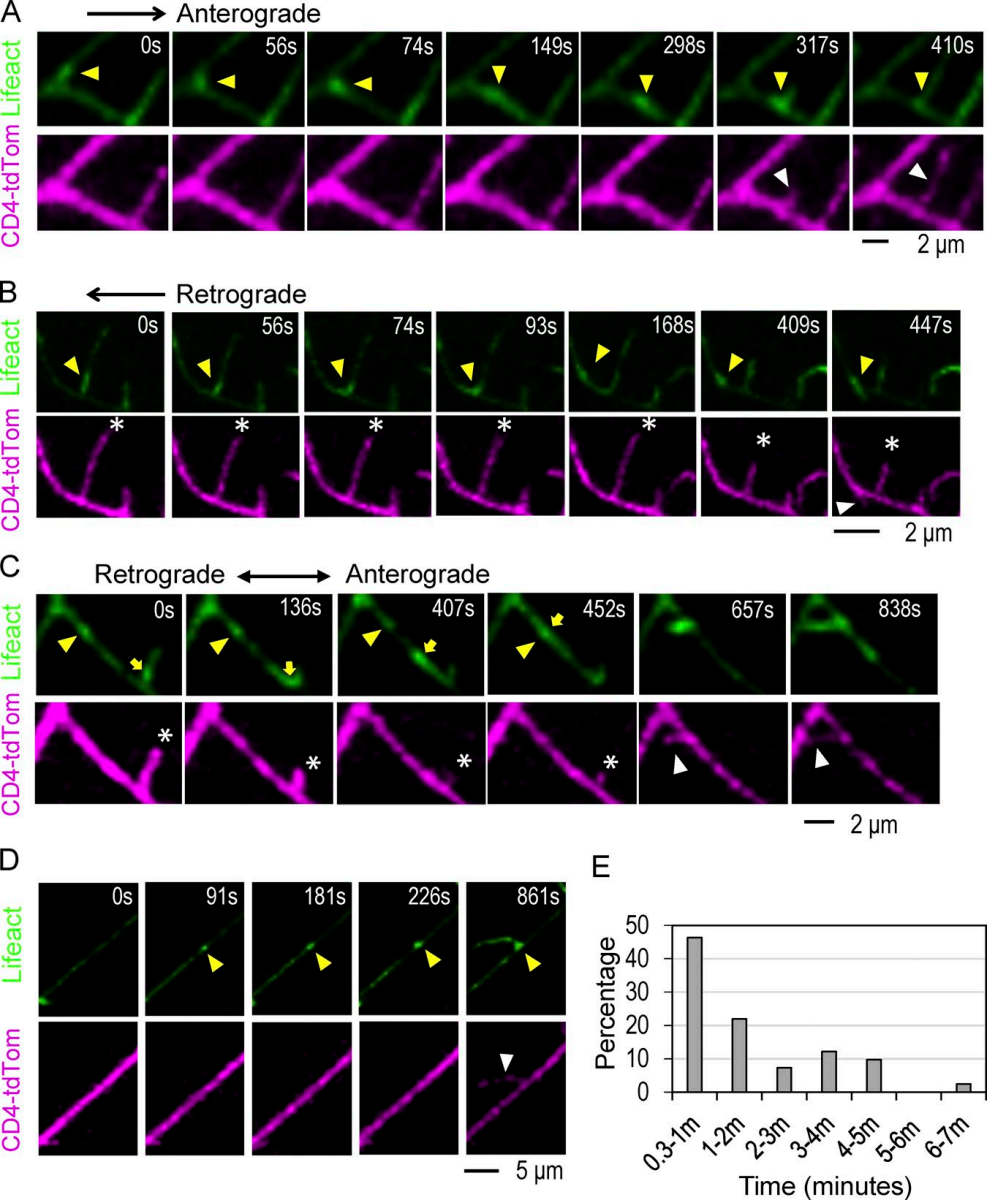
**Actin blob prelocalization to future branching sites. (A)** Time series of images show the movement of an actin blob (green; indicated by yellow arrowheads) in anterograde direction (0–149 s), stalling (298 s), and the emergence of a new branch (317 s) indicated by white arrowheads with dendrites labeled in magenta (Video 6). **(B)** The actin blob moves out of a retracting dendrite (0–93 s) and stalls (168–409 s) until the emergence of new dendrite (447 s). The retracting dendrite is marked with asterisks, and a newly emerging dendrite is marked by a white arrowhead (Video 7). **(C)** Two actin blobs (one indicated by yellow arrowheads, and one by yellow arrows) merge at the stalling site (452 s) to induce dendrite emergence (white arrowhead at 657 s and 838 s). The retracting dendrite is marked with asterisks (Video 8). **(D)** F-actin accumulation (yellow arrowheads in 91–226 s) until a new dendrite emerges (white arrowhead at 861 s; Video 9). **(E)** Bar graph shows the distribution of actin blob stalling times before branch emergence from 41 stalling/branching events in 10 neurons from seven experiments.

To explore possible actin blob functions in dendrites, we sought any correlation between actin blob dynamics and morphogenetic processes such as the formation of new branches and extension and retraction of existing branches. Interestingly, when following actin blob propagation, we found that actin blobs stalled at sites where new branches would bud out soon after (Fig. 2, A–C; and Videos 6, 7, and 8). A striking correlation was detected; almost all new branches (83% in LifeAct- and 87.5% in GMA-marked actin blobs; see Table 2 for sample numbers hereafter) had a prelocalized actin blob at the branching site. Prior to the prelocalization, 27.3% of actin blobs anterogradely propagated and 54.5% retrogradely propagated to the branching sites (Fig. 2, A and B; Videos 6 and 7; and Table 2 hereafter). In the remaining 18.2% of cases, two actin blobs approached each other and stalled together at the branching site (Fig. 2 C and Video 8). Interestingly, retrograde actin blobs derived from neighboring retracting dendrites contributed to new branch formation (Fig. 2, B and C). In the absence of actin blob prelocalization, new branches emerged with local F-actin accumulation (Fig. 2 D and Video 9; 11.3%) or even without enriched F-actin at the branching site (5.7%). The time from actin blob localization to branch initiation could range from <20 s to >7 min, with the majority of actin blobs prelocalized for <2 min (Fig. 2 E). On average, new dendrites had actin blobs prelocalized for 1 min 54 s before branching out. The level of actin blob intensity after localization was maintained or fluctuated slightly until branching, suggesting that specific changes such as increases in the F-actin level are not prerequisite for branching. Once new branches emerged, we observed the infusion of F-actin into the new dendrite, which might indicate the growth of F-actin (Fig. 2, A–D).

In class IV da neurons, 24% of prelocalized actin blobs led to a branch formation, while the rest dispersed after the stalling (Table 2). When stalled for >1 min, the percentage leading to branch formation increased to 50%. To exclude the possibility that actin blob localization in branching dendrites is stochastic, we examined the correlation between the actin blob localization site and the dendrite branching site. In the 24 actin blobs we assayed, 17 (71%) of them localized within 1 µm of the branching site in a 10-µm dendrite, and seven (29%) localized to nonbranching region (P < 0.0001 for random association by χ^2^ test). Indeed, in those colocalized cases, the distance between the center of the actin blob and the center of the new branching site was merely 0.16 µm on average, suggesting a tight correlation between these two processes.

As dynamic terminal dendrites contained higher numbers of actin blobs, we assayed a possible correlation between actin blob propagation and dendrite dynamics. During dendrite retraction, 33.3% dendrites had a retrograde actin blob, 9.5% had an anterograde actin blob, 23.8% were devoid of actin blobs, and the remaining 33.3% had two actin blobs moving in the opposite direction. During dendrite extension, a higher percentage of dendrites (57.1%) had a retrograde actin blob as compared with retracting dendrites. Dendrites with an anterograde actin blob remained the same (9.5%), while dendrites containing no actin blobs (14.3%) or bidirectional actin blobs (19.1%) were reduced. With retrograde actin blobs accounting for the highest percentages in both extending and retracting dendrites, it seems that directionality of actin blob propagation does not dictate changes in terminal dendrite dynamics.

A previous study indicates actin accumulation at the branching site precedes the branching event in class III da neurons (Andersen et al., 2005). To address whether the presence of actin blobs in class IV da neurons is neuronal type specific, we examined LifeAct signals in class III da neurons. Consistently, in those new branching events recorded in 10 min within a 10-µm dendrite, the number of actin blobs was significantly reduced to 0.6 as compared with 2.5 per in class IV da neurons (Table 1). Also, actin blob prelocalization accounted for ∼35% of new dendrite formation as compared with >80% of new dendrite formation in class IV da neurons (Table 2). However, while actin blobs were the minor event in class III da neurons, they shared comparable properties with actin blobs in class IV da neurons including velocity (1.8 µm/min in class III vs. 1.4 µm/min in class IV; P = 0.03 by Student’s *t* test) and percent stalled events leading to branching (70% in class III vs. 71% in class IV; P = 0.496 by proportion test). Taken together, these analyses suggest that the branching mechanism by actin blob prelocalization is also used in class III da neurons but as a minor pathway.

### F-actin–stabilizing G15S mutant reduces actin blobs and dendrite dynamics

**Figure 3.**
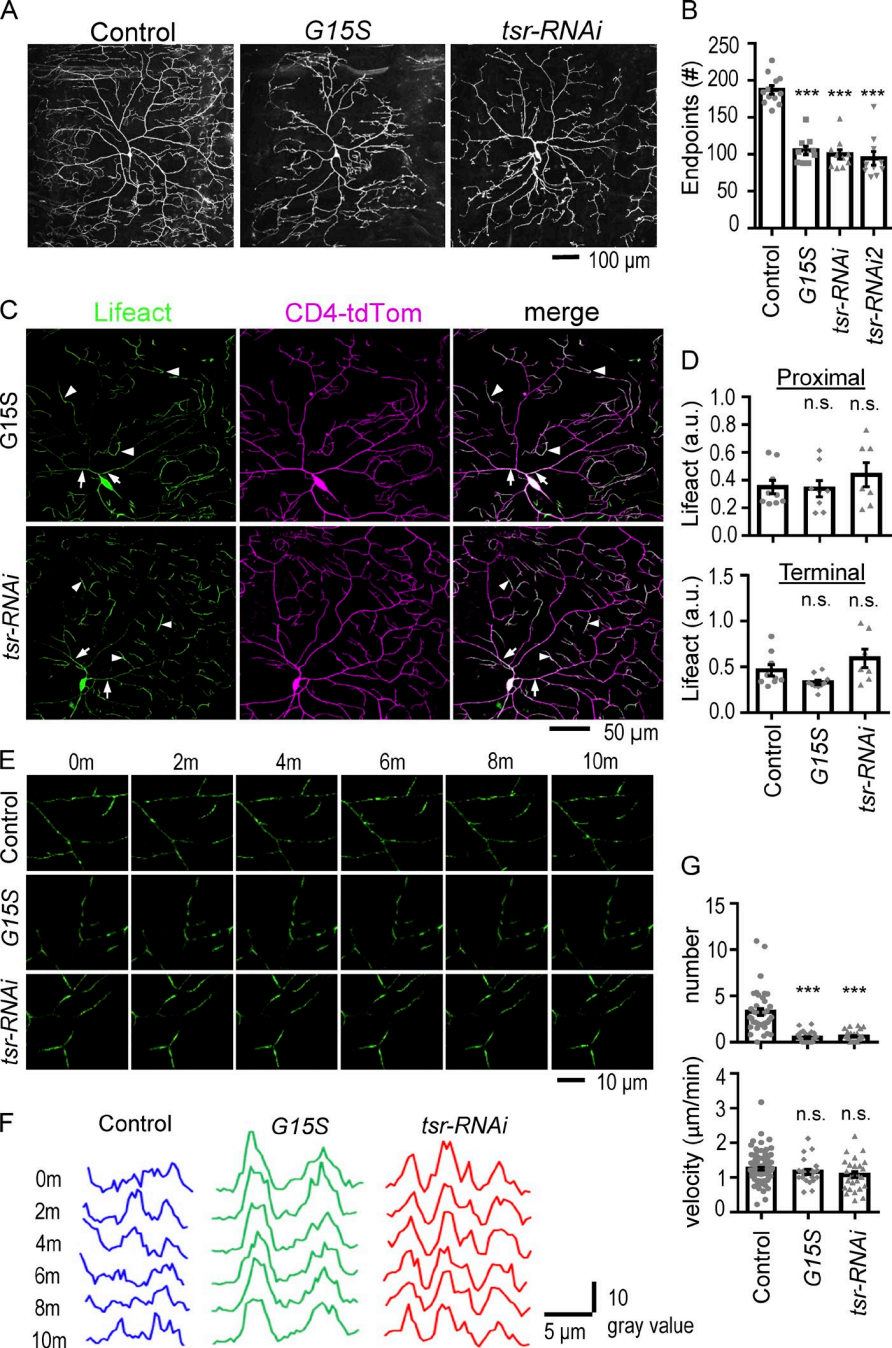
**F-actin distribution and dynamics in *G15S* and *tsr-RNAi* neurons. (A)** Images show dendritic trees in control (*ppk-GAL4/+; ppk-CD4-td-Tom/+*), *G15S*-expressing, and *tsr-RNAi*–knockdown neurons. **(B)** Bar graph shows average numbers of dendritic endpoints present in the posterior dorsal region of the ddaC dendritic field. The *tsr-RNAi2* effect is also included. Numbers of neurons: 12 (control), 10 (*G15S*), 10 (*tsr-RNAi*)*,* and 10 (*tsr-RNAi2*). **(C)** Images show LifeAct distributions (green) in the CD4-tdTomato–labeled dendrites (magenta) of *G15S*-expressing (top) and *tsr-RNAi*–knockdown (bottom) neurons. Merged images of two channels are at right. Arrows indicate high LifeAct signals in proximal dendrites, and arrowheads indicate these in terminal dendrites. For control, see Fig. 1 A. **(D)** Bar graphs show quantifications of LifeAct intensities normalized to CD4-tdTomato intensities in proximal dendrites (top) as indicated by arrows in C as well as terminal dendrites (bottom) as indicated by arrowheads in C. Each dot represents the average value from one neuron. Numbers of neurons: 9 (control), 8 (*G15S*), and 7 (*tsr-RNAi*) from five to six experiments. **(E)** LifeAct signals are dynamic (Video 10) in control terminal dendrites (top) and remain static in both *G15S-*expressing (middle) and *tsr-RNAi*–knockdown (bottom) terminal dendrites. Images are shown in time series of frames separated by 2-min intervals for a total of 10 min. **(F)** Line graphs show changes of LifeAct intensities in representative segments of dendrites over a 10-min live-imaging period with 2-min intervals for control (blue), *G15S*-expressing (green), and *tsr-RNAi*–knockdown (red) neurons. The intensities were assayed from linearized dendrites. x axis, μm; y axis, LifeAct gray value. Representative images were chosen from observations in 39 dendrites for control, 28 dendrites for *tsr-RNAi*, and 39 dendrites for *G15S* in six neurons from six experiments for each genotype. **(G)** Bar graphs show quantifications for actin blob numbers per 10 µm recorded in 10 min (top) as well as actin blob velocity in μm/min (bottom) in control (94 actin blobs in 39 dendrites), *G15S-*expressing (23 actin blobs in 29 dendrites), and *tsr-RNAi*–knockdown (26 actin blobs in 28 dendrites) neurons from six neurons in six experiments for each genotype. Genotypes for control: *UAS-lacZ-RNAi/+; ppk-GAL4, UAS-LifeAct/+; ppk-CD4-td-Tom/+*; for *G15S*: *+/+; ppk-GAL4, UAS-LifeAct/UAS-ActinG15S; ppk-CD4-td-Tom/+*; and for *tsr-RNAi*: *+/+; ppk-GAL4, UAS-LifeAct/UAS-tsr-RNAi; ppk-CD4-td-Tom/+*. Significance in comparison with control was determined by Student’s *t* test. ***, P < 0.001. Error bars represent SEM.

The actin mutant G15S can be incorporated into F-actin that is more stable than F-actin constituted of solely WT actin (Posern et al., 2004). To study how stabilization of F-actin might have an impact on actin blobs and dendrite morphogenesis, the *UAS-G15S* transgene was expressed in class IV da neurons. We first showed that in *G15S*-expressing neurons, the dendritic pattern was dramatically compromised, with the number of endpoints greatly reduced (Fig. 3, A and B; and Table 3 for averages and SD), suggesting that overstabilized F-actin compromised dendrite growth. When G15S was coexpressed with LifeAct, the overall F-actin distribution remained similar to the pattern in control arbor (compare Fig. 3 C with Fig. 1 A). Higher levels of F-actin were present in proximal and terminal dendrites as in control (Fig. 3 C, arrows and arrowheads, respectively). Quantification showed no significant difference in comparing F-actin intensities (Fig. 3 D). Strikingly, the F-actin signals were static when examined in live imaging in contrast with WT control dendrites in which F-actin signals were highly dynamic (Fig. 3, E and F; and Video 10). Hence, actin blobs, defined as dynamic F-actin assemblies, were drastically reduced in *G15S*-expressing neurons. In control neurons, 97.4% of dendrites had at least one actin blob when recorded within a 10-µm segment in 10 min compared with 55.2% of dendrites in *G15S*-expressing neurons. The average number of actin blobs in a 10-µm dendrite was also greatly reduced by the *G15S* expression, from 3.3 in control to 0.5 in *G15S*-expressing neurons, a more than sixfold reduction (Fig. 3 G, top). Consistent with the reduction of actin blobs in dendrites, the actin blob splitting events were also greatly reduced to 7.1% in *G15S-*expressing neurons, also a sixfold reduction from 42.1% in control. The actin blob size was also reduced to 1.8 µm compared with 3.1 µm in the control neurons (Table 3). Unexpectedly, those dynamic actin blobs that remained in *G15S*-expressing neurons propagated at a comparable speed of 1.2 µm/min (Fig. 3 G, bottom). Taken together, these results show that overstabilization of F-actin by G15S mainly reduces the number of actin blobs rather than the speed of propagation in dendrites.

**Table 3.** Effect of *G15S* expression and *tsr* knockdown on dendrites

Description	**Average ± SD (sample number)**			
	**Control**	**Act42A**	***G15S***	***tsr-RNAi***
Dendrites / c4ddaC (number)	187 ± 20 (*n* = 12)	163 ± 37 (*n* = 9)	105 ± 18 (*n* = 10)	100 ± 20 (*n* = 10)
Dendrites / c3ddaF (number)	263 ± 51 (*n* = 15)	239 ± 41 (*n* = 9)	64 ± 12 (*n* = 10)	142 ± 21 (*n* = 10)
Dendrites / c3ddaA (number)	329 ± 50 (*n* = 10)	331 ± 60 (*n* = 9)	72 ± 19 (*n* = 9)	181± 48 (*n* = 8)
Dendrites / c1ddaE (MARCM; number)	25 ± 6.6 (*n* = 11)	ND^a^	ND	19.6 ± 2.9 (*n* = 10)
Dendrites / c1ddaE (number)	23.8 ± 2.7 (*n* = 13)	22.2 ± 3.1 (*n* = 8)	12.6 ± 1.8 (*n* = 9)	ND
LifeAct intensity in proximal dendrites (AU)	0.4 ± 0.1 (*n* = 9)	0.4 ± 0.1 (*n* = 6)	0.3 ± 0.2 (*n* = 8)	0.4 ± 0.2 (*n* = 7)
LifeAct intensity in terminal dendrites (AU)	0.5 ± 0.2 (*n* = 9)	0.5 ± 0.1 (*n* = 6)	0.3 ± 0.1 (*n* = 10)	0.6 ± 0.3 (*n* = 7)
Dendrites with actin blobs (%)	97.4 (*n* = 39)	93.3 (*n* = 15)	55.2 (*n* = 29)	67.9 (*n* = 28)
Actin blobs in terminal dendrites (in 10 µm)	3.3 ± 2.3 (*n* = 39)	2.8 ± 1.8 (*n* = 15)	0.5 ± 0.6 (*n* = 29)	0.6 ± 0.6 (*n* = 28)
Dendrites with splitting events (%)	42.1 (*n* = 38)	33.3 (*n* = 15)	7.1 (*n* = 14)	0 (*n* = 28)
Velocity in terminal dendrites (μm/min)	1.3 ± 0.4 (*n* = 94)	1.4 ± 0.5 (*n* = 61)	1.2 ± 0.4 (*n* = 23)	1.1 ± 0.5 (*n* = 26)
Actin blob size (μm)	3.1 ± 1.1 (*n* = 68)	3.1 ± 1.4 (*n* = 22)	1.8 ± 0.8 (*n* = 20)	2.0 ± 0.9 (*n* = 23)
New dendrites (in 10^4^ μm^2^)	12.5 ± 7.4 (*n* = 14)	12.2 ± 8.8 (*n* = 6)	1.9 ± 2.3 (*n* = 12)	3.1 ± 4.5 (*n* = 11)
New dendrites with prelocalization (%)	83.0 (*n* = 53)	78.9 (*n* = 19)	33.3 (*n* = 18)	47.4 (*n* = 19)
Normalized LifeAct intensity in new dendrites (AU)	1.0 ± 0.4 (*n* = 30)	1.0 ± 0.2 (*n* = 18)	0.5 ± 0.3 (*n* = 21)	0.6 ± 0.2 (*n* = 22)
New dendrite growth in 5 min (μm)	3.8 ± 1.9 (*n* = 25)	4.0 ± 1.4 (*n* = 20)	2.5 ± 0.9 (*n* = 14)	2.3 ± 0.9 (*n* = 13)
Existing dendrite extension in 10 min (μm)	3.3 ± 0.5 (*n* = 81)	3.4 ± 1.3 (*n* = 20)	1.1 ± 0.2 (*n* = 48)	1.9 ± 0.5 (*n* = 62)
Existing dendrite retraction in 10 min (μm)	3.1 ± 0.9 (*n* = 81)	3.8 ± 0.7 (*n* = 20)	1.3 ± 0.4 (*n* = 48)	2.0 ± 0.5 (*n* = 62)

a Not done.

**Figure 4.**
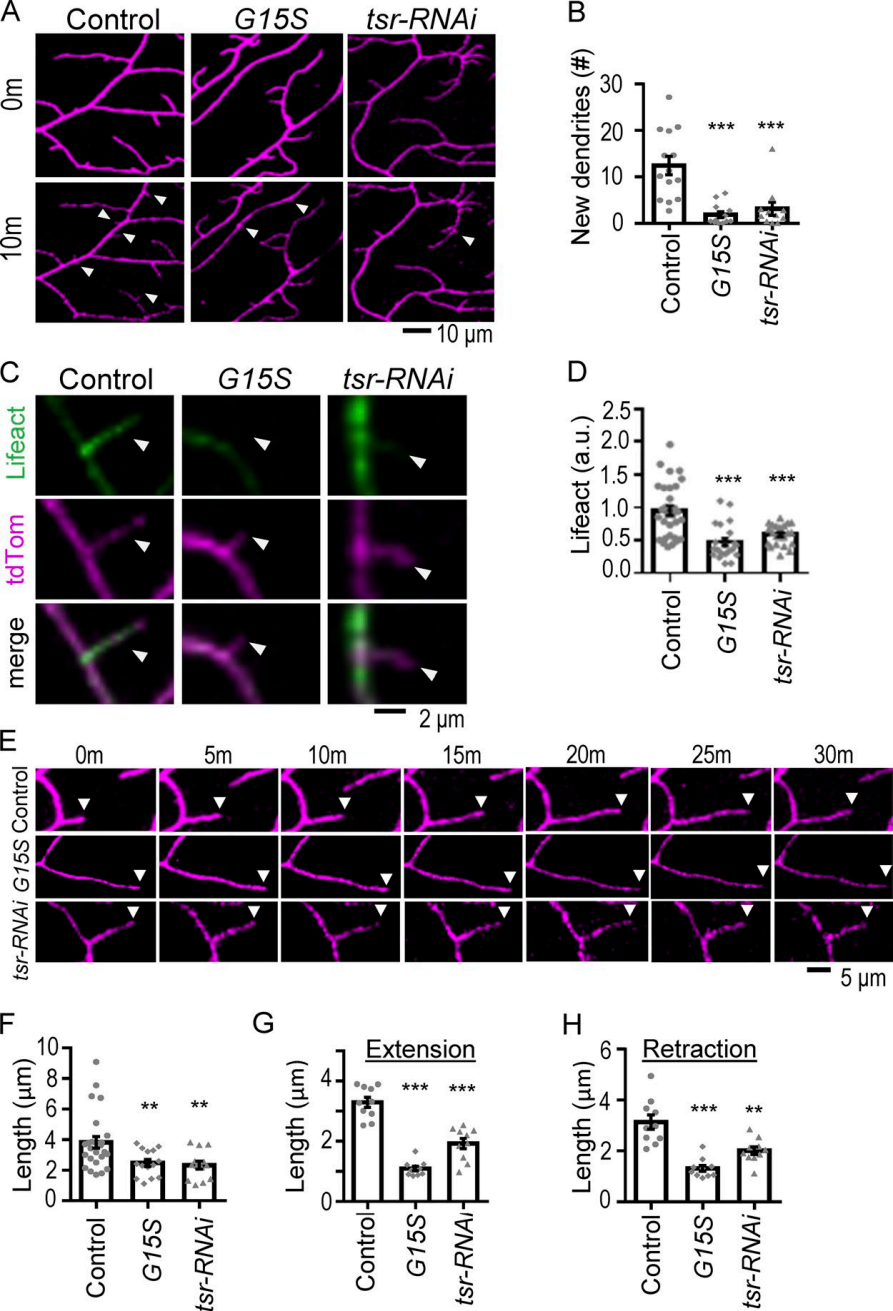
**Dendrite formation and motility in *G15S* and *tsr-RNAi* neurons. (A)** Images show new dendrite formation in control, *G15S-*expressing, and *tsr-RNAi*–knockdown neurons. Arrowheads indicate the sites at which new buds have emerged. The images at the beginning (0 min) and the end (10 min) of the observation are shown. **(B)** Bar graph indicates the average numbers of new dendrites per 10,000 µm^2^ of the dorsal posterior field. The dendritic field was live imaged for 10 min with 20-s intervals for each scan. New dendrites that had emerged during this period were scored. Numbers of neurons are 14 for control, 12 for *G15S*, and 11 for *tsr-RNAi* from 7 to 10 experiments. **(C)** Images show LifeAct signals (green, top) in new branches (magenta, middle and merge images at bottom) that emerged within 10 min live imaging for control (left), *G15S-*expressing (middle), and *tsr-RNAi*–knockdown (right) neurons. **(D)** Bar graph represents LifeAct intensities (normalized to CD4-tdTomato intensities) in new branches. Numbers of branches are 30 for control, 21 for *G15S*, and 22 for *tsr-RNAi* from seven neurons in 10 experiments for each genotype. **(E)** Time series of images show dendrite dynamics in 30 min with 5-min intervals. Arrowheads indicate dendritic tips to show dendrite dynamic growth. **(F–H)** Bar graphs show average length in tip displacement of new dendrites for 5 min (F) as well as extension (G) and retraction (H) of existing dendrites for 10 min in control, *G15S-*expressing, and *tsr-RNAi*–knockdown neurons. In F, numbers of dendrites are 25 (control), 14 (*G15S*), and 13 (*tsr-RNAi*) from six to eight neurons in four to five experiments. In G and H, number of dendrites are 81 (control), 48 (*G15S*), and 62 (*tsr-RNAi*) from 10 neurons in seven to eight experiments for each genotype, and each dot represents the average number for a neuron. Significance in comparison with control was determined by Student’s *t* test. **, P < 0.01; ***, P < 0.001. Error bars represent SEM.

With a sixfold reduction in the number of actin blobs, whose localization marked the future dendrite branching sites, we examined whether new dendrite branching is also affected in *G15S*-expressing neurons. While in control neurons, 12.5 new buds emerged in an area of 10,000 µm^2^ in 10 min, only 1.9 were detected in *G15S*-expressing neurons, a more than sixfold reduction that could resonance the reduction in actin blobs (Fig. 4, A and B). Although the numbers of new branches and actin blobs were both reduced by sixfold, 33.3% of branching events in *G15S*-expressing neurons still had prelocalized actin blobs before branching out. These analyses are consistent with the idea that F-actin stabilization by G15S hinders the production of actin blobs, which results in the reduced availability for branching out new dendrites.

Furthermore, during the emergence of new branches in *G15S*-expressing neurons, F-actin infusion into the new dendrites was lacking, resulting in dramatic reduction of LifeAct signals in new dendrites (Fig. 4, C and D). The reduction of F-actin correlated with slow growth of these new dendrites. The net displacement of new dendrites in 5 min was 3.8 µm in control neurons, whereas it was 2.5 µm for *G15S*-expressing neurons (Fig. 4 F). In addition, mature dendrites often undergo constant extension and retraction. We found that both extension and retraction were retarded in *G15S*-expressing neurons (Fig. 4 E). In the 10-min recording, control dendrites had an extension of 3.3 µm and a retraction of 3.1 µm, whereas *G15S*-expressing dendrites had an extension of 1.1 µm and a retraction of 1.3 µm (Fig. 4, G and H). Therefore, overstabilization of F-actin in *G15S*-expressing neurons causes slower growth of new dendrites and reduced motility of existing dendrites.

Maintaining dynamic F-actin is likely essential for all types of neurons during dendrite arborization. We examined whether G15S-overstabilized F-actin had impacts on branching in other types of da neurons (Fig. S3). As expected, G15S expression resulted in the reduction of filopodia-like protrusions in class III da neurons (Table 3). Also, the class I da neuron with simple arbor was also compromised in branching (Table 3). Thus, F-actin overstabilization had adverse effects on the growth of da dendrites we had examined.

To test whether the effect of G15S could be due to increased levels of actin, the WT version of actin, *Act42A*, was overexpressed by the *ppk-GAL4* driver. The branching pattern, terminal dendrite dynamics, F-actin intensities, and actin blob dynamics were all indistinguishable to control neurons (Fig. S4 and Table 3). Thus, the effects of G15S on actin blobs and dendrites are caused by actin overstabilization rather than the consequence of actin overexpression.

### Tsr/cofilin regulates actin blobs and dendrite branching

**Figure 5.**
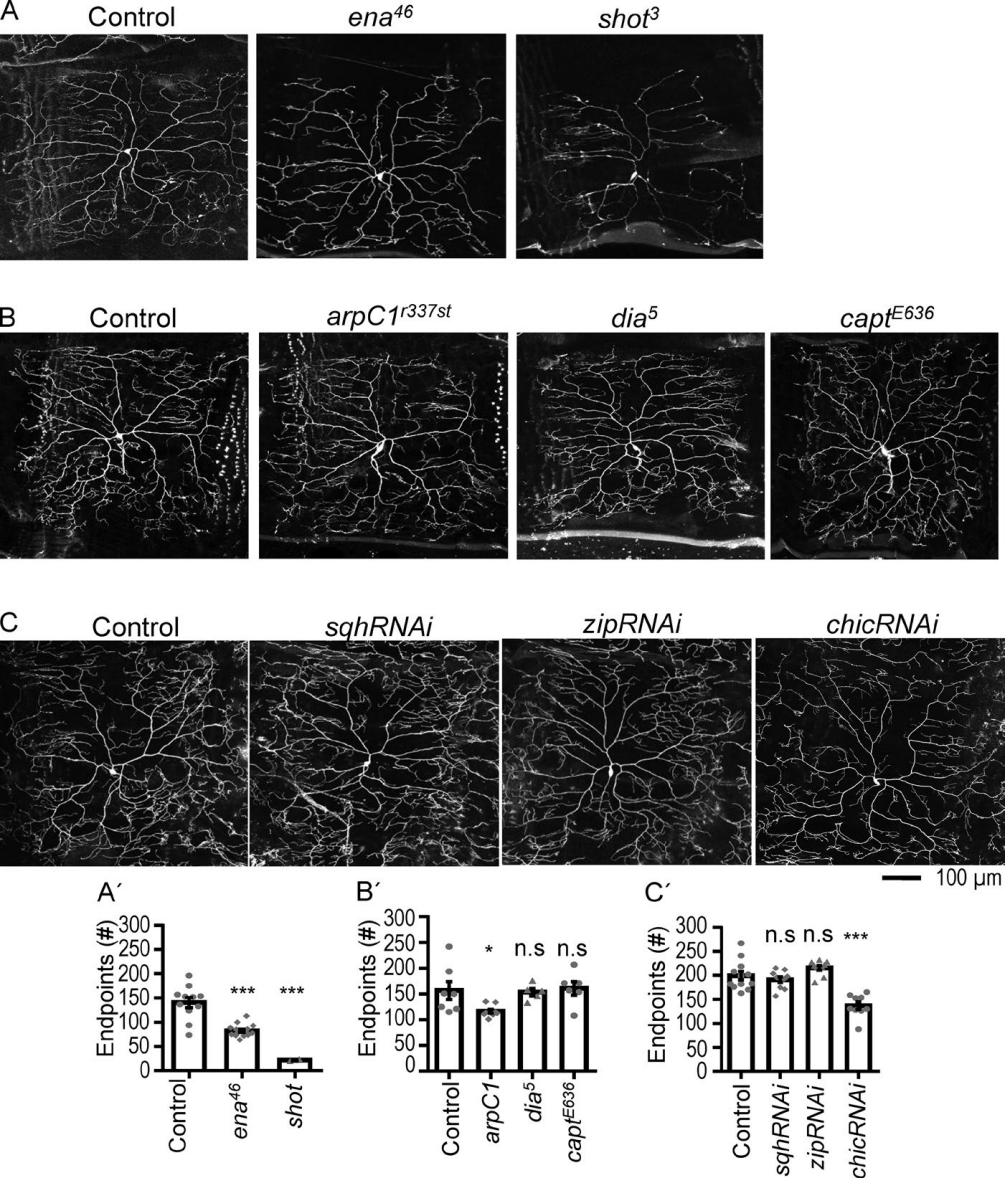
**Dendritic phenotypes in mutants for actin regulatory proteins. (A and B)** MARCM clones were generated for *ena^46^* (*n* = 15 neurons) and *shot^3^* (*n* = 2) as compared with control *FRT^G13^* (*n* = 11; A) as well as *arpC1^r337st^* (*n* = 8), *dia^5^* (*n* = 5), and *capt^E636^* (*n* = 6) as compared with control *FRT^40A^* (*n* = 7; B). **(C)** RNAi knockdowns for *sqh* (*n* = 10), *zip* (*n* = 10), and *chic* (*n* = 9) were compared with *ppk-GAL4* control (*n* = 12). Bar graphs in A′–C′ show the number of dendritic endpoints for A–C, respectively, scored in the dorsal posterior region of class IV da neurons at the wandering third instar stage. Each dot represents the number of endpoints in a neuron. *shot*^3^ exhibited reduced availability of MARCM clones, indicating neuronal loss. Significance was determined using Student’s *t* test. *, P < 0.05; ***, P < 0.001. Error bars represent SEM.

To screen regulators of actin blobs, several proteins that are known to regulate different aspects of actin structure and function were tested. Initially, mosaic analysis with a repressible cell marker (MARCM) clones or RNAi knockdown were employed to examine dendritic defects in mutants for these F-actin regulators (Figs. 5 and S5). While defective dendritic patterns were found in some of the F-actin regulators, we chose Tsr/cofilin for further study since Tsr/cofilin might be involved in actin blob production through severing F-actin (Zebda et al., 2000). Also, cofilin was unable to bind G15S-stabilized F-actin (Posern et al., 2004), prompting us to examine whether *tsr* mutant neurons present similar defects.

**Figure 6.**
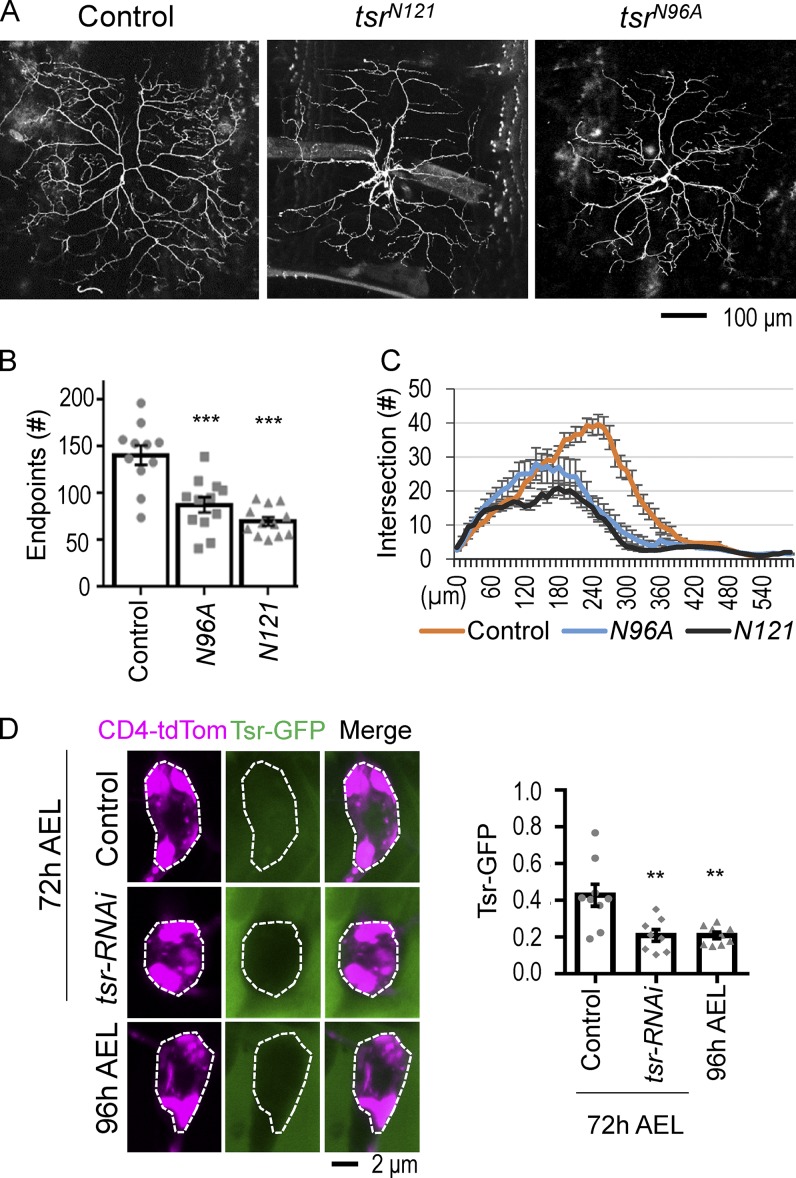
**Dendrite morphology in *tsr* mutants. (A)** Defective ddaC dendritic patterns in MARCM clones for *FRT^G13^ tsr^N121^* (*n* = 12) and *FRT^G13^ tsr^N96A^* (*n* = 12) marked by *GAL4^5-40^*-driven *UAS-Venus* for labeling dendrites, with *FRT^G13^ tsr*^+^ (*n* = 11) used as control MARCM. **(B)** Bar graph shows dendritic endpoints in the posterior dorsal region of ddaC neurons. The control (*FRT^G13^* MARCM) is a replicate of the control in Fig. 5 A′. **(C)** Sholl analysis shows dendrite intersections with concentric rings in 10-µm increments from the proximal region until the distal end. Number of neurons: 9 (control), 11 (*tsr^N121^*), and 12 (*tsr^N96A^*) from two to three independent experiments. **(D)** Immunointensity of Tsr-GFP in class IV da neuron at 72 h (*n* = 9) and 96 h AEL (*n* = 9) and in *tsr-RNAi* (*n* = 8) neurons at 72 h AEL from two to three experiments. Tsr-GFP intensities normalized to CD4-tdTomato within soma (outlined by dashed lines) were scored and shown in the bar graph (right). Tsr-GFP intensity was normalized to the neuronal marker. Significance was determined using Student’s *t* test. **, P < 0.01; ***, P < 0.001. Error bars represent SEM. Each dot represents a neuron.

MARCM clones for *tsr^N121^* or *tsr^N96A^*, both loss-of-function alleles, exhibited a drastic defect in dendrite branching throughout the long primary and secondary dendrites (Fig. 6 A). Quantification of dendritic ends showed a strong reduction of branches (Fig. 6 B), and Sholl analysis indicates that branch reduction was more pronounced in medial and distal regions of the arbor (Fig. 6 C). To further confirm the role of Tsr in dendrite branching, two different *tsr-RNAi* lines that strongly reduced *tsr* mRNA levels (Fig. S5 G) were expressed in class IV da neurons. The numbers of dendritic ends were also reduced in the *tsr* knockdown neurons as compared with the control (Fig. 3, A and B). As shown for G15S overexpression, we also showed that the dendritic patterns of class I and class III da neurons were defective in *tsr* MARCM clones or by *tsr-RNAi* knockdown (Fig. S3 and Table 3). Altogether, these analyses suggest that the F-actin–severing protein Tsr/cofilin is required for dendrite branching in different classes of da neurons.

Using a protein trap line in which *tsr* is fused to *GFP* to examine Tsr expression during development, we found that Tsr was expressed at higher levels at 72 h than at 96 h AEL (Fig. 6 D), consistent with the timing when actin blobs were dynamic (Fig. 1 J). Similar to the previous observation (Nowak et al., 2010; Grintsevich et al., 2016), Tsr-GFP signal is present throughout the cell body. Expressing *tsr-RNAi* reduced Tsr-GFP signal in the class IV da neuron (Fig. 6 D). To explore the possible role of Tsr in regulating actin blobs, we observed LifeAct in dendrites with *tsr-RNAi* knockdown. The distribution of LifeAct signals appeared to be comparable with control dendrites, with higher levels of intensities in proximal and terminal dendrites (arrows and arrowheads, respectively, in bottom panels of Fig. 3 C, and quantification in Fig. 3 D). Consistent with *G15S*-expressing neurons, LifeAct-enriched signals were static without dynamic movement (Fig. 3, E and F; and Video 10). We tracked for dynamic actin blobs in dendrites of *tsr-RNAi* neurons. The number of dendrites including at least one actin blob in 10-µm dendrites was reduced from 97.4% of control dendrites to 67.9% of *tsr-RNAi* dendrites. The average number of actin blobs per 10-µm terminal dendrite was greatly reduced by *tsr-RNAi* knockdown (control, 3.3; *tsr-RNAi*, 0.6; Fig. 3 G, top). Actin blob size reduced from 3.1 µm in control to 2 µm in *tsr-RNAi* dendrites (Table 3). Similar to *G15S* expression, *tsr-RNAi* knockdown did not affect the actin blob velocity significantly (Fig. 3 G, bottom). Thus, Tsr regulates mainly the availability of actin blobs in dendrites.

The 5.5-fold reduction in the number of actin blobs also resulted in the reduction of new dendrites in *tsr-RNAi* neurons. In an area of 10,000 µm^2^, only 3.1 new branches were detected in 10-min imaging of *tsr-RNAi* neurons (Fig. 4, A and B), a fourfold reduction from control neuron. New dendrites emerged from *tsr-RNAi* neurons still had prelocalized actin blobs (47.4%) before branching. Thus, similar to *G15S*-expressing neurons, Tsr is specifically required for the production of actin blobs in dendrites.

Furthermore, during the emergence of new branches in *tsr-RNAi* neurons, F-actin failed to infuse into new dendrites, resulting in dramatic reduction in LifeAct intensities (arrowheads in Fig. 4 C and quantification in Fig. 4 D). The reduction of F-actin in new dendrites correlated with slow dendrite growth in *tsr-RNAi* neurons with reduced displacement of dendritic tips (Fig. 4 F). The constant extension and retraction of existing dendrites were also compromised in 10 min of recording (Fig. 4, E, G, and H). Therefore, the F-actin dynamics were reduced in *tsr-RNAi* neurons, resulting in slower dendrite growth and motility.

We further clarified whether dendrite growth defects could have a general effect on F-actin motility and actin blob availability. In the screening of actin regulators, we also identified Ena and Chic/profilin, which were required for normal dendrite branching (Fig. 5, A and C). By studying Ena and Chic, known to be involved in actin polymerization, and monomeric actin pool maintenance, we could examine their roles on actin blob properties. Overexpression of dominant-negative *ena-DN* (Lebrand et al., 2004) also compromised dendritic arbor (Fig. S5, A and B; and Table S1). Interestingly, the distribution of LifeAct signals in *ena-DN* and *chic-RNAi* neurons remained in a pattern comparable with that in the control, with high levels in the proximal and terminal dendrites (Fig. S5 C). These actin blobs were also highly dynamic in dendrites (Fig. S5 D), and the number of actin blobs showed no significant difference with control (Fig. S5 E). Actin blob size was reduced in *chic-RNAi* dendrites (Table S1). However, the velocities of actin blobs were slightly increased in *ena-DN* and *chic-RNAi* neurons (Fig. S5 F). Hence, Chic and Ena might regulate an alternative aspect rather than actin blobs in dendrite branching.

Next, we tested the role of the cyclase-associated protein Capulet, which sequesters monomeric G-actin and dissociates Cofilin from ADP-bound actin (Hubberstey et al., 1996; Moriyama and Yahara, 2002). As indicated in the previous study, *capt^E636^* MARCM clones do not show any dendrite-branching defect (Medina et al., 2008), which was further confirmed by *capt-RNAi* knockdown (Figs. 5 B and S5, A and B). Actin distribution and actin blob dynamics in *capt-RNAi*–knockdown dendrites were comparable with the control with a slight increase in velocity (Fig. S5, C–F; and Table S1). It shows that Capulet is not essential for dendrite branching or actin blob propagation. The comparison of these mutant phenotypes singles out the specificity of the actin-severing protein Tsr in actin blob regulation and F-actin dynamic in class IV da dendrites.

## Discussion

In summary, we have identified an actin population in dendrites, which we named actin blobs. These dynamic actin blobs propagate bidirectionally in dendrites and, when stalled, mark future dendrite branching sites. The F-actin–severing protein Tsr/cofilin is involved in the regulation of actin blobs. In *tsr* knockdown, actin blobs were reduced, and dendrite branching was compromised. The *tsr* knockdown recapitulated dendritic and actin blob phenotypes observed in *G15S* overexpression that induced F-actin stabilization, suggesting that the dynamics of F-actin are crucial in actin blob production and dendrite branching. Hence, through exploring the function and regulation of actin blobs, we propose a new mechanism for the involvement of F-actin in dendrite branching.

### Actin blob dynamics in dendrites

The dynamic actin blobs in growing dendrites have distinct properties compared with actin trails and waves. Actin trails elongate at a much faster speed of 1 µm/s in axons (Ganguly et al., 2015). Actin blobs in our study propagated at a slower rate of 1 µm/min. Actin trails elongate at one end due to polymerization, leaving an elongated trail behind, while actin blobs moved as an entity without associated trails or comets to the moving blobs. The trail movement in dendrites has not been reported. Actin waves, another dynamic entity in neurons, emerge from the base and propagate toward the end of the growing neurites, and they move only in the anterograde direction (Ruthel and Banker, 1999). Actin blobs originated from various parts of the dendrites even from terminal dendrites and moved bidirectionally. While propagation of actin waves depends on microtubules, actin blob movements were normal when microtubules were largely interrupted by RNAi depletion or Katanin-mediated severing (Fig. S1). Also, the functions of actin blobs characterized in dendrites are very different to actin waves and trails. Actin waves propagated to the axonal or dendritic ends are likely to promote continuing growth, and actin trails might be involved in synaptic recycling. We propose that actin blobs are involved in dendrite branching. However, actin blobs shared certain characteristics with actin waves. Both move at a velocity of ∼1 µm/min in dendrites (Ruthel and Banker, 1999). Similar to actin waves, actin blob dynamics varied depending on the developmental stage. Actin waves are frequently observed in the early developmental stages and diminished in the later stages (Flynn et al., 2009). Similarly, the number of dynamic actin blobs was higher in the neurons of early third instar larvae, the stage at which dendrites are highly dynamic (Stewart et al., 2012). In late larval stages when dendrite growth is prohibited, LifeAct signals appeared static, and dynamic actin blobs were not detected (Fig. 1 J). In dendrites of class IV da neurons, actin rod-like structures and Spire-nucleated F-actin structures were observed in fixed neurons (Medina et al., 2008; Ferreira et al., 2014). Whether they share similar properties to actin blobs and their dynamic properties remains to be known. Taken together, dynamic F-actin structures with distinct characteristics indicate that multitier organization of actin could contribute to various cellular needs in neurons.

### Actin blob localization at future dendrite branching sites

F-actin–based filopodial protrusion from the dendritic shaft underlies the initial event in dendrite branching and spine formation (Andersen et al., 2005; Korobova and Svitkina, 2010; Hou et al., 2015). The branching sites accumulate F-actin before dendrite initiation, and enriched F-actin extends into the growing dendrites. One mechanism of F-actin accumulation is initiated by the actin nucleation factor Cobl (Hou et al., 2015). Localization of Cobl precedes F-actin accumulation at the branching site, which is followed by the dendrite emergence. In cultured neurons, Cobl responds to Ca^2+^ signaling through the Ca^2+^-sensing protein calmodulin (CaM), which interacts with and activates Cobl, leading to the growth of actin filaments. Cobl also interacts with the F-bar protein syndapin to coordinate membrane outgrowth. In our study, F-actin accumulation at the branching site was detected only in a small proportion of dendrites (Fig. 2 D). Rather than initiating F-actin formation in situ, dynamic actin blobs moved to and localized at the future branching sites, a process preceding branch initiation. We failed to detect a role of syndapin in dendrite formation in class IV da neurons, and the correlation between Ca^2+^ signaling and actin blob localization was not evident, indicating a fundamentally distinct mechanism in F-actin enrichment at the branching site. Indeed, in dendrites of class III da neurons, actin locally aggregates before formation of actin-rich filopodia, which could be promoted by activated CaMKII (Andersen et al., 2005). Interestingly, similar manipulation by overexpression of activated CaMKII failed to induce filopodia in class IV da neurons (Andersen et al., 2005). In class III da neurons, we observed that 35% of the dendritic spike-forming events had actin blob localization, while the majority of new dendrites (83%) in class IV da neurons had prelocalized actin blobs. By analyzing these two types of da neurons in *Drosophila*, it is clear that different types of neurons employ distinct mechanisms for actin enrichment at the branching site. These differences might be attributed to various types of neurons that grow their dendrites in different patterns or respond to different environmental cues. Considering the huge diversities of neurons, further unidentified mechanisms are expected.

### Tsr/cofilin regulates dendrite branching

In this study, we also focused on finding proteins involved in actin blob regulation. By morphology-based genetic screening, we had found that mutations in *ena*, *chic*, and *tsr* caused severe dendrite defects, and we further analyzed their roles in actin blob dynamics. Tsr/cofilin, an F-actin–severing and –depolymerizing protein, regulates the population of dynamic actin blobs in dendrites. In the absence of Tsr/cofilin, most of the actin blobs were static, while dynamic blobs, although much fewer, propagated at the normal speed as in control dendrites (Fig. 3 G). The defect in *tsr* mutants thus could not be explained simply by a disruption in actin polymerization, which would lead to a compromise in the speed of propagation. To support this idea, we had analyzed the *ena* mutant that is supposed to compromise actin polymerization (Barzik et al., 2005). In the *ena* mutant, while dendrite patterning was affected, the number of actin blobs and their dynamics were normal. Alternatively, depleting the monomeric actin pool in the *tsr* mutants could have an impact on actin blob dynamics. Chic/profilin through recycling ADP-actin to ATP-actin maintains a monomeric F-actin pool (Goldschmidt-Clermont et al., 1991). We had also analyzed mutants for Chic and found no defect in actin blob numbers and dynamics. Instead, *tsr* knockdown and *G15S* expression, which is resistant to cofilin binding, caused a drastic reduction in the number of dynamic actin blobs, suggesting that the F-actin–severing activity contributes to the generation of actin blobs. Consistently, the actin blob–splitting event that might depend on the severing activity of cofilin was drastically reduced in both *tsr* knockdown and *G15S* expression (Table 3). Interestingly, in both *tsr* and *chic* knockdown neurons, the actin blob size was reduced. Both Chic/profilin and Tsr/cofilin are important to maintain the monomeric actin pool in vivo, and the reduction in monomeric actin might influence the actin blob size (Table S1). So far, the forces propelling the actin blob movement in dendrites remain unclear.

The role of cofilin in the regulation of dendritic spines has been studied previously (Gu et al., 2010; Noguchi et al., 2016). During long-term potentiation, cofilin is required during the initial phase of spine enlargement but is inactivated during stabilization (Fukazawa et al., 2003; Bosch et al., 2014). Also, cofilin is involved in spine shrinkage (Zhou et al., 2004). However, cofilin regulation on dendrite branching remained elusive. Earlier studies have ascertained that mechanisms of spine regulation are different from those regulating dendrite branches (Haas et al., 2013; Copf, 2016). Interestingly, in contrast with the role of cofilin in dendrite branching shown in our study, loss of cofilin results in an increase in the spine density (Rust et al., 2010). Also, membrane protrusion mediated by the inverse BAR protein MIM is a prior event than actin enrichment in spine initiation (Saarikangas et al., 2015). We failed to detect a dendritic defect in RNAi knockdown of the *Drosophila* MIM protein in class IV da neurons. Thus, distinct mechanisms involving cofilin are evident between spine and dendrite formation. In support of a role of Tsr in regulating actin blobs during dendrite growth, higher levels of Tsr-GFP expression were detected in early to mid–third instar larvae, when both actin blobs and dendrite branching are still highly active (Fig. 6 D). We propose that Tsr/cofilin-regulated dynamic actin blobs play an important role in shaping dendrite architecture.

## Materials and methods

### Fly stocks

GAL4 lines used for restricted expressions in this study were *ppk-GAL4* for class IV da neurons (Kuo et al., 2005), *GAL4^19-12^* for class III da neurons (Xiang et al., 2010), *IG1-1-GAL4* for class I da neurons (Sugimura et al., 2003), *da-GAL4* for ubiquitous expression (Wodarz et al., 1995), and *GAL4^5-^*^40^ for all peripheral sensory neurons (Song et al., 2007). *ppk-CD4-tdTomato* for labeling class IV da neurons (Han et al., 2011) was obtained from the Bloomington Drosophila Stock Center (BDSC). Mutant flies used in this study were *arpC1* (9136; Hudson and Cooley, 2002), *dia^5^* (9138; Castrillon and Wasserman, 1994), *capt^E636^* (5944; Benlali et al., 2000), *tsr^N121^* (9109; Ng and Luo, 2004), and *tsr^N96A^* (9108; Ng and Luo, 2004) from BDSC; *shot^3^* (108072; Kolodziej et al., 1995) from the Kyoto Stock Center; and *ena^46^* (Gao et al., 1999; Gates et al., 2009). Flies carrying RNAi transgenes were *UAS-zip-RNAi* (36727; He et al., 2014), *UAS-chic-RNAi* (34523; Kooij et al., 2016), and *UAS-tsr-RNAi* (65055; Grintsevich et al., 2016) from the BDSC; *UAS-sqh-RNAi* (7916; Majumder et al., 2012), *UAS-tsr-RNAi2* (110599; Abe et al., 2014), *UAS-capt-RNAi* (21995; Marrone et al., 2011), and *UAS-αTub84B-RNAi* (33427) from the Vienna Drosophila Resource Center; and *UAS-lacZ-RNAi* (Kennerdell and Carthew, 2000). The Ena dominant-negative form targeting to mitochondria was expressed by *UAS-FLAG-HA-FP4mito* (58481; BDSC). The GFP trap *tsr* line *tsr^CPTI002237^* (115280; Grintsevich et al., 2016) was from Kyoto Stock Center. Other transgenes used were *UAS-Jupiter-mCherry* (Cabernard and Doe, 2009), *UAS-myc-Actin G15S*, and *UAS-myc-Act42A* (Hsiao et al., 2014). *UAS-LifeAct-GFP* (35544; Hatan et al., 2011), *UAS-LifeAct-RFP* (58362; Cai et al., 2014), *UAS-Kat60* (64115; Mao et al., 2014), and *UAS-GMA* (31775 and 31774; Kiehart et al., 2000) were obtained from BDSC.

### Immunostaining

Larvae were dissected and fixed in 4% paraformaldehyde for 30 min and washed thrice with PBST. After blocking in 5% normal donkey serum for 2 h, larvae fillets were incubated in primary antibody overnight followed by PBST wash. After incubation in secondary antibody for 2 h, fillets were washed in PBST and mounted in glycerol followed by imaging by a LSM 710 (ZEISS) microscope with C-Apochromat 40× 1.2 W Korr objective lens. Primary antibodies used were mouse anti–α-tubulin (B512; 1:200; Sigma-Aldrich) and mouse anti-Futsch (22C10; 1:100; Developmental Studies Hybridoma Bank). Alexa Fluor 488– and Cy5-conjugated secondary antibodies are from Jackson ImmunoResearch Laboratories, Inc.

### RT-PCR

*da-GAL4* was used to drive *RNAi* expression in the larvae. As knocking down *sqh*, *zip*, *tsr*, and *chic* led to lethality after first instar stage, RNA was extracted from first instar larvae. In case of *capt-RNAi*, RNA was extracted from wandering third instar larvae. RNA was converted to cDNA using SuperScript IV VILO from Invitrogen, and PCR was performed with corresponding primers, with *RpL19* expression serving as the internal control. Expression levels are compared with *da-Gal4* crossed with *w^1118^*. Primer pairs used were *RpL19*, 5′-TCTCTAAAGCTCCAGAAGAGGC-3′ and 5′-CGATCTCGTTGATTTCATTGGGA-3′; *sqh*, 5′-CGAGGAGAATATGGGCGTCC-3′ and 5′-CCTCCCGATACATCTCGTCCA-3′; *zip*, 5′-CCAAGACGGTCAAAAACGAT-3′ and 5′-GATGTTGGCTCCCGAGATAA-3′; *chic*, 5′-ATGAGCTGGCAAGATTATGTGG-3′ and 5′-TCCTCTTTTGTCACCTCAAAGC-3′; *tsr*, 5′-GCTCTCAAGAAGTCGCTCGT-3′ and 5′-GCAATGCACAGTGCTCGTAC-3′; and *capt*, 5′-GTCCGCTGAGCCAATACCTAA-3′ and 5′-CAAAGGCGCTCTTCACGAG-3′.

### Image acquisition and processing

Live imaging was performed as per the previously described protocol with modifications (Lin et al., 2015). Flies expressing LifeAct under *ppk-GAL4* with *ppk-CD4-tdTomato* were crossed with corresponding flies containing other UAS transgenes. Eggs were collected for 4 h in yeast-containing fly food vials. After 20 h, hatched larvae were removed to start timing. Early third instar larvae (72 ± 4 h AEL) were used for imaging LifeAct and CD4-tdTomato in class IV da neurons. For studying the mid–third instar larvae, identical protocol was followed, and larvae were imaged at 96 ± 4 h AEL. Larvae were fixed alive on a double-sided sticky tape in a slide and covered with a coverslip. The inverted confocal microscopes LSM 880 and 710 (ZEISS) with C-Apochromat 40× 1.2 W Korr objective lens were used for imaging. Imaging was done at the room temperature with Immersol W (ZEISS) between lens and coverslip. Live imaging was performed for 10–30 min with 20-s intervals on average using Zen software (ZEISS). Spinning-disk confocal microscopes (CSU-X1; ZEISS; and Revolution WD; Andor Technology) were also used for imaging with similar experimental setup. We used a Nikon Ti-E microscope with an Andor Ultra 888 charge-coupled device. The objective lens used was Plan Apochromat lambda 60× 1.4 NA oil. Images were acquired using MetaMorph software (Molecular Devices). Live imaging in a spinning-disk microscope was performed for 10 min with 1-s intervals on average. Images from larvae that were still alive and active after imaging were taken for further analysis. Larvae with neurons showing dendrite varicosities were not taken for analysis. Fluorescent proteins used in this study were GFP, YFP, RFP, mCherry, and tdTomato. While processing the images and videos, brightness and contrast were adjusted and Gaussian smoothing was performed using Zen 2012 software. Video 1 was generated using Imaris (8.3.1; Bitplane).

### Data analysis

#### Actin blob identification and measurement

Propagating actin blobs were followed in live images of LifeAct in Zen 2012 software. Kymographs were performed using MetaMorph software (version 7.6) to follow the actin blobs in which each horizontal line represents a single time point and the change in position of F-actin intensity over time indicates the propagation of actin blobs. While performing kymographs, average background intensity was subtracted. Actin blobs that were dynamic in live images and had signal intensity shift in the kymograph were taken for velocity measurement in MetaMorph. F-actin that grows or retreats at the dendrite tip along with the change in dendrite length were excluded. Hence, F-actin clusters that move inside the dendritic shafts are regarded as actin blobs.

#### Measuring LifeAct intensity

For measuring F-actin intensities, dendrites were straightened using ImageJ (National Institutes of Health), and LifeAct intensity was normalized to CD4-tdTomato intensity. LifeAct intensity in new branches was taken when the new dendrite reached the maximum length during the period of observation and was normalized to CD4-tdTomato intensity.

#### Counting dendrite numbers

Dendrite endpoints were measured in wandering late third instar larvae. Larvae were immobilized in water kept on ice for 10 min to reduce larval crawling. Afterwards, they were mounted dorsal side up on a slide with a drop of glycerol and crushed using a coverslip. Dendrites were imaged in LSM 710 using a Plan Apochromat 20× 0.8 objective lens. Dendrite endpoints were counted manually in the dorsal posterior region of the class IV da neuron. For class I and class III da neurons, dendrite endpoints of the entire neuron were counted.

#### Measuring actin blob prelocalization to branching sites

New dendrites that showed increased levels of LifeAct at the branching site compared with neighboring regions before branching event were considered as a prelocalization event. Stall actin blobs at the branching sites were traced back for their movements in retrograde, anterograde, or both directions. When the LifeAct intensity at the future branching site accumulated locally without prior propagation to the branching site, they were added to the percentage of dendrites without actin blob prelocalization. Branching events that had F-actin localized at the future branching site from the beginning of the observation were excluded from the analysis.

#### Correlating actin blob propagation to dendrite extension and retraction

Existing dendrites that showed continuous extension or retraction for ≥3 min were taken for analysis. Directionality of actin blob propagation near the dendrite tip before or during the extension or retraction process were noted. F-actin that grows or retreats along with the change in dendrite length was excluded.

#### Measuring new dendrite numbers

Newly formed dendrites were tracked in the dorsal posterior region for 30 frames in a total of 10 min. New dendrite numbers were normalized to the dendritic field area of 10,000 µm^2^.

#### Measuring dendrite extension and retraction

The existing dendrites at the beginning of imaging were taken for analysis. Change in dendrite length was measured for 10 min once every 20 s on average. Lengths of dendrite extension and retraction were calculated for each dendrite and averaged per neuron.

#### Measuring new dendrite growth

The frame that a newly branched out dendrite was spotted was defined as the time 0. Dendrite length was measured after 5 min from the time 0, representing the dendrite growth in 5 min.

#### Statistical methods

Student’s *t* tests were performed to determine whether the difference between the test and control groups was statistically significant. χ^2^ tests were used to compare the observed and expected values in actin blob prelocalization to the branching site. Proportion tests were used to compare the actin blob prelocalization in class IV and class III da neurons. Comparisons of data between control and mutants in bar graphs are shown as averages and SEM with asterisks indicating statistical significance by Student’s *t* test: *, P < 0.05; **, P < 0.01; ***, P < 0.001. For providing original data and their distributions, we have used SD in all tables.

### Online supplemental material

Fig. S1 shows dynamic actin blobs in microtubules disrupted neurons. Fig. S2 shows GMA-probed actin blobs in dendrites. Fig. S3 shows dendritic phenotypes of class I and III da neurons by *G15S* overexpression or *tsr* depletion. Fig. S4 shows F-actin dynamics in WT actin–overexpressing neurons. Fig. S5 shows phenotypes in Ena dominant-negative, *chic-RNAi*, and *capt-RNAi* neurons. Table S1 shows screening for dendrite and actin blob regulators. Video 1 shows actin blob propagation in the retrograde direction. Video 2 shows anterograde movement of an actin blob. Video 3 shows passage of an actin blob through a branching site. Video 4 shows actin blob splitting. Video 5 shows GMA propagating in dendrites. Video 6 shows an anterogradely propagating actin blob seeding a new dendrite branch. Video 7 shows a retrogradely propagating actin blob seeding a new dendrite branch. Video 8 shows two populations of actin blobs seeding a new dendrite. Video 9 shows local actin growth before dendrite branching. Video 10 shows F-actin dynamics in dendrites of control, *G15S*, and *tsr-RNAi*.

## Supplementary Material

Supplemental Materials (PDF)

Video 1

Video 2

Video 3

Video 4

Video 5

Video 6

Video 7

Video 8

Video 9

Video 10
