# Supplementary material for: Actin blobs prefigure dendrite branching sites
Source: J Cell Biol. 2018 Oct 1;217(10):3731–46. doi: 10.1083/jcb.201711136 (PMC6168249; doi:10.1083/jcb.201711136)
Supplement: Supplemental Materials (PDF) [file JCB_201711136_sm.pdf]

Supplemental material

Nithianandam and Chien, https://doi.org/10.1083/jcb.201711136

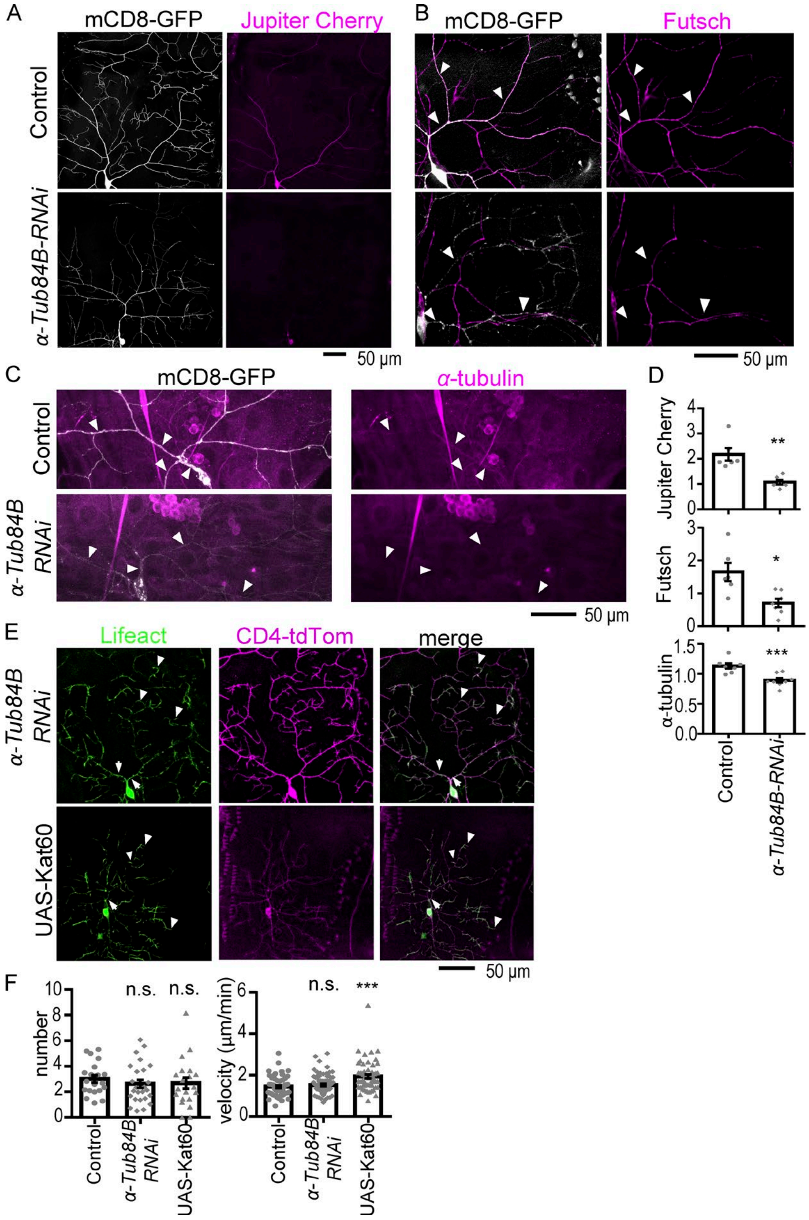

Figure S1. **Dynamic actin blobs in disrupted microtubules. (A–C)** Reduced microtubules in α*Tub84B-RNAi* neurons. **(A)** Expression of microtubule-associated Jupiter-Cherry (magenta; right) was reduced in α*Tub84B-RNAi* (bottom) driven by *ppk-GAL4* in dendrites marked by mCD8-GFP (left). **(B and C)** Microtubule reduction was also shown by immunostaining for Futsch (B) and α-tubulin (C). Arrowheads indicate class IV da dendrites. **(D)** Bar graphs with dots (representing neurons) show immunostaining intensities for Jupiter-Cherry (top), Futsch (middle), and α-tubulin (bottom). Jupiter-Cherry intensity was normalized to background. Futsch intensity was normalized to dendrites of neighboring neurons. α-Tubulin intensity was normalized to neighboring tissues. Numbers of neurons for Jupiter-Cherry were six in control and *αTub84B-RNAi*; for Futsch, they were six in control and seven in *αTub84B-RNAi*; and for α-tubulin, they were seven in control and eight in *αTub84B-RNAi*. **(E)** Images show α*Tub84B-RNAi* knockdown (top) and *UAS-Kat60* expression (bottom) for LifeAct, CD4-tdTomato, and merge channels. Arrows and arrowheads indicate proximal and terminal dendrites, respectively. **(F)** Comparing numbers (left) and velocities (right) of actin blobs in *ppk-GAL4* control, *αTub84B-RNAi*–, and *UAS-Kat60*–expressing neurons. Each dot in left graph represents the numbers of actin blobs per 10 µm recorded within 10 min, and in the right graph, each dot represents the velocity of an actin blob. Note a slight increase in the actin blob velocity in *Kat-60*–expressing neurons. In total, 52 actin blobs in 20 dendrites for control from three neurons in one experiment (in addition to similar observations in Fig. 1 I), 87 actin blobs in 25 dendrites for *αTub84B-RNAi* from five neurons in two experiments, and 58 actin blobs in 20 dendrites for *UAS-Kat60* from five neurons in two experiments. Significance was determined using Student's *t* test. *, P < 0.05; **, P < 0.01; ***, P < 0.001. Error bars represent SEM.

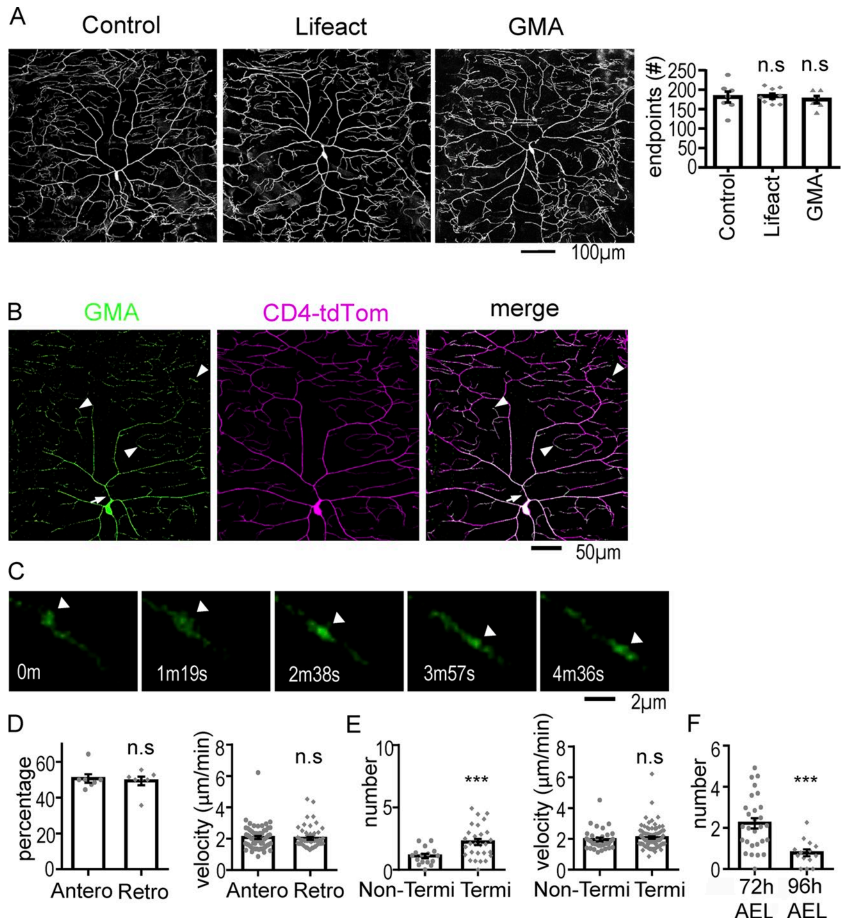

Figure S2. **GMA-probed actin blobs in dendrites. (A)** Images show normal dendritic patterns of class IV da neurons in *ppk-GAL4* control (n = 7) or expressing LifeAct (n = 9) or GMA (n = 6). Bar graph shows average numbers of dendritic endpoints present in the posterior dorsal region of the ddaC dendritic field. Each dot represents a neuron. **(B)** Distribution of GMA (green) expressed by *ppk-GAL4* in dendritic arbor of class IV da neurons marked by *ppk-CD4-tdTomato* (magenta). Arrows indicate high GMA signals in proximal dendrites, and arrowheads indicate these in terminal dendrites. **(C)** Time series images show GMA-marked actin blob propagation in anterograde direction (see also Video 5). **(D–F)** Properties of GMA-marked actin blobs. **(D)** Bar graphs compare percentages (n = 7 neurons for both) and velocities of GMA-marked actin blobs between anterograde (Antero; n = 63) and retrograde (Retro; n = 58) propagation. **(E)** Comparing actin blob numbers per 10 µm (left) and velocities (µm/min; right) in nonterminal (Non-termi; 33 actin blobs in 16 dendrites) and terminal (Termi; 88 actin blobs in 28 dendrites) dendrites. **(F)** Comparing GMA-marked actin blob numbers in 10-µm terminal dendrites in early third instar (72 h AEL; replicate of terminal dendrites in E) and mid–third instar (96 h AEL; n = 14) neurons. Significance was determined using Student's t test. ***, $P < 0.001$. Error bars represent SEM.

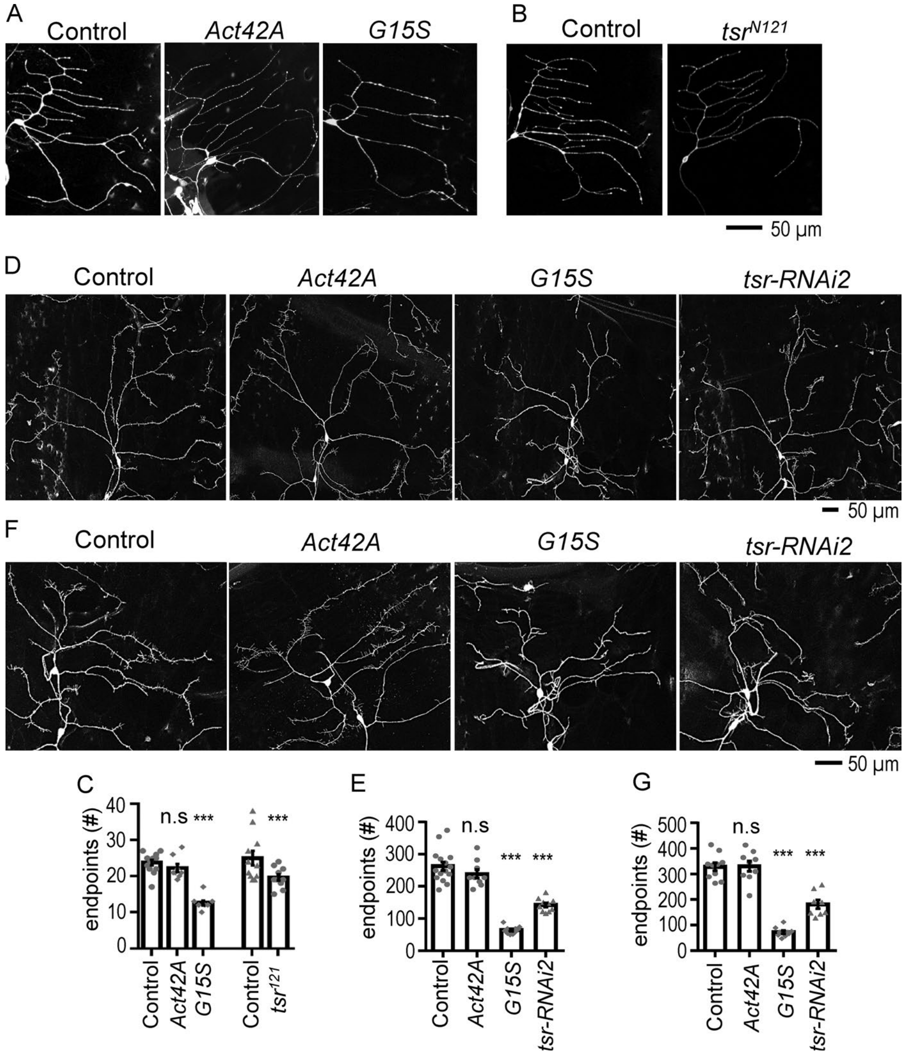

Figure S3. **Dendritic phenotypes of class I and III da neurons by *G15S* overexpression or *tsr* depletion. (A)** Images show *IG1-1-GAL4*–driven *UAS-mCD8-GFP* marking class I ddaE dendrites in control, *Act42A*-expressing, and *G15S*-expressing neurons. **(B)** Images show class I da neurons in FRT^{G13} control and tsr^{N121} MARCM mutant clones marked by $GAL4^{5\text{-}40}$-driven *UAS-Venus*. **(C)** Bar graph shows quantification of dendritic endpoints in class I da neurons. **(D and F)** Images show dendritic spikes in class III ddaF (D) and ddaA (F) of control, *Act42A*-expressing, *G15S*-expressing, and *tsr-RNAi2*–knockdown neurons. **(E and G)** Bar graphs show quantification of dendritic spikes in ddaF and ddaA neurons (see Table 3 for average numbers). Number of neurons in C: 13 (control), 8 (*Act42A*), 9 (*G15S*), 11 (FRT^{G13} control), and 10 (tsr^{N121}); numbers in E: 15 (control), 9 (*Act42A*), 10 (*G15S*), and 10 (*tsr-RNAi2*); numbers in G: 10 (control), 9 (*Act42A*), 9 (*G15S*), and 8 (*tsr-RNAi2*). Significance was determined using Student's *t* test. ***, P < 0.001. Error bars represent SEM.

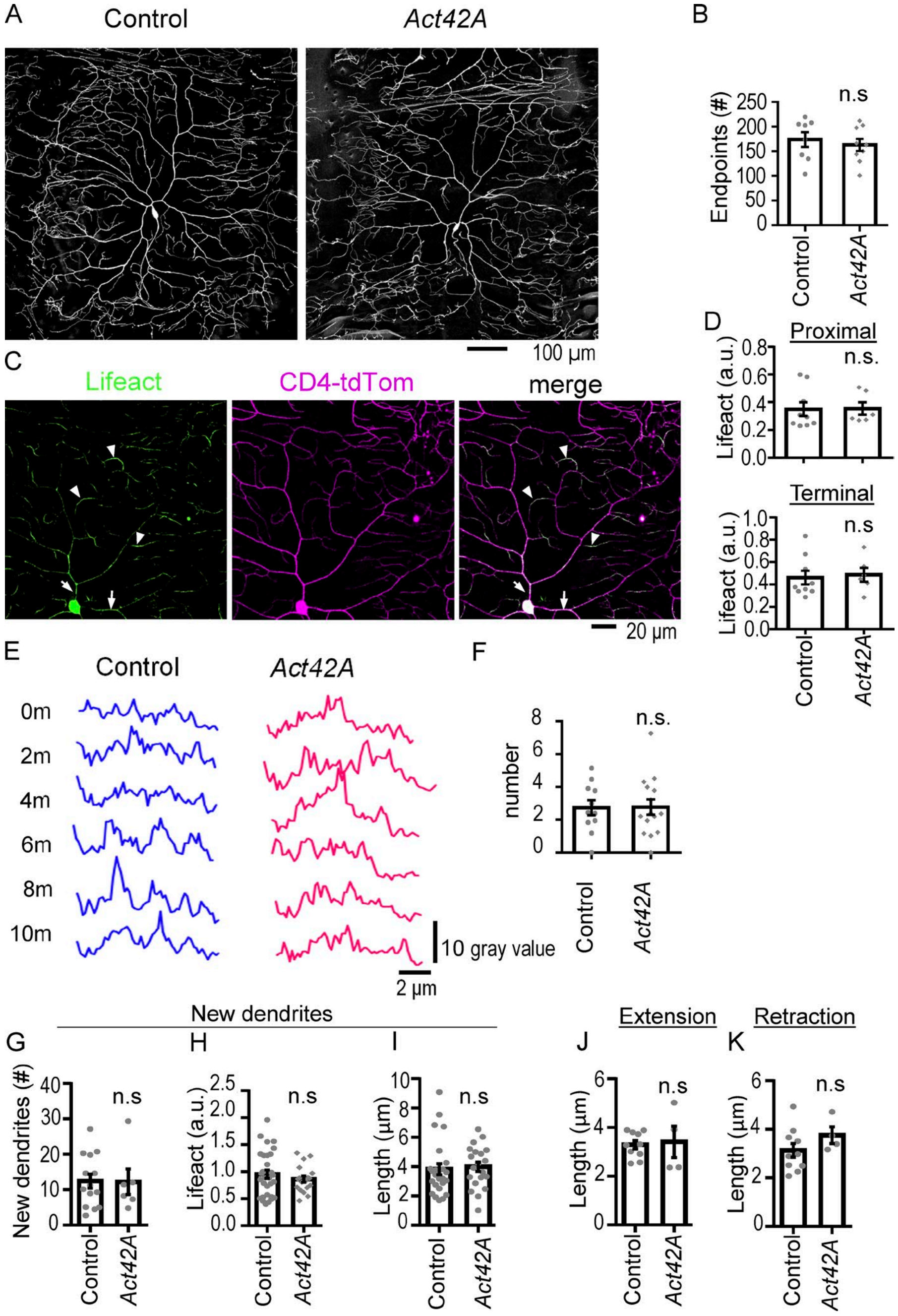

Figure S4. **F-actin dynamics in overexpression of WT actin. (A)** Images show dendritic trees in control (*ppk-GAL4/+; ppk-CD4-td-Tom/+*) and *Act42A*-expressing neurons (*ppk-GAL4/+; ppk-CD4-td-Tom/UAS-myc-Act42A*). **(B)** Bar graph shows average numbers of dendritic endpoints present in the posterior dorsal region of the ddaC dendritic field in *Act42A*-expressing neurons compared with control. Numbers of neurons were eight (control) and nine (*Act42A*). **(C)** Images show LifeAct distributions (green) in the CD4-tdTomato–labeled dendrites (magenta) of *Act42A*-expressing neurons. Merged images of two channels are at right. Arrows indicate high LifeAct signals in proximal dendrites, and arrowheads indicate these in terminal dendrites. **(D)** Bar graphs show quantifications of LifeAct intensities normalized to CD4-tdTomato intensities in proximal (top) and terminal (bottom) dendrites. Each dot represents the average value from one neuron. Numbers of neurons were 9 (control) and 6 (*Act42A*). **(E)** Line graphs show changes of LifeAct intensities in control (blue) and *Act42A*-expressing (pink) dendrites over a 10-min live-imaging period with 2-min intervals for each line. The intensities were assayed from linearized dendrites (x axis, μm in length; y axis, gray value). Representative images were chosen from 11 dendrites of two neurons in control and from 15 dendrites from four neurons in *Act42A* from two experiments. **(F)** Bar graphs show quantifications for actin blob numbers per 10 μm in 10 min in control (*n* = 11 dendrites; in addition to similar observations in Fig. 1 I) and *Act42A*-expressing (*n* = 15) terminal dendrites. **(G)** Bar graph indicates the average numbers of new dendrites per 10,000 μm^2 of the dorsal posterior field emerged in 10 min from 14 neurons in control and six in *Act42A*. **(H)** Bar graph represents LifeAct intensities (normalized to CD4-tdTomato intensities) in 30 new branches in control and 18 in *Act42A*. **(I–K)** Bar graphs show average length in tip displacement of new dendrites in 5 min (I) as well as extension (J) and retraction (K) of existing dendrites for 10 min in control and *Act42A*-expressing neurons. Numbers of dendrites in I were 25 (control) and 20 (*Act42A*), and number of neurons in J and K were 10 (control) and 4 (*Act42A*K). In total, three to five *Act42A*-expressing neurons from two experiments were recorded. Significance in comparison with control was determined by Student's *t* test. Error bars represent SEM. Controls of *ppk-GAL4* driver in B, D, and G–K are the same as in respective Figs. 3 and 4.

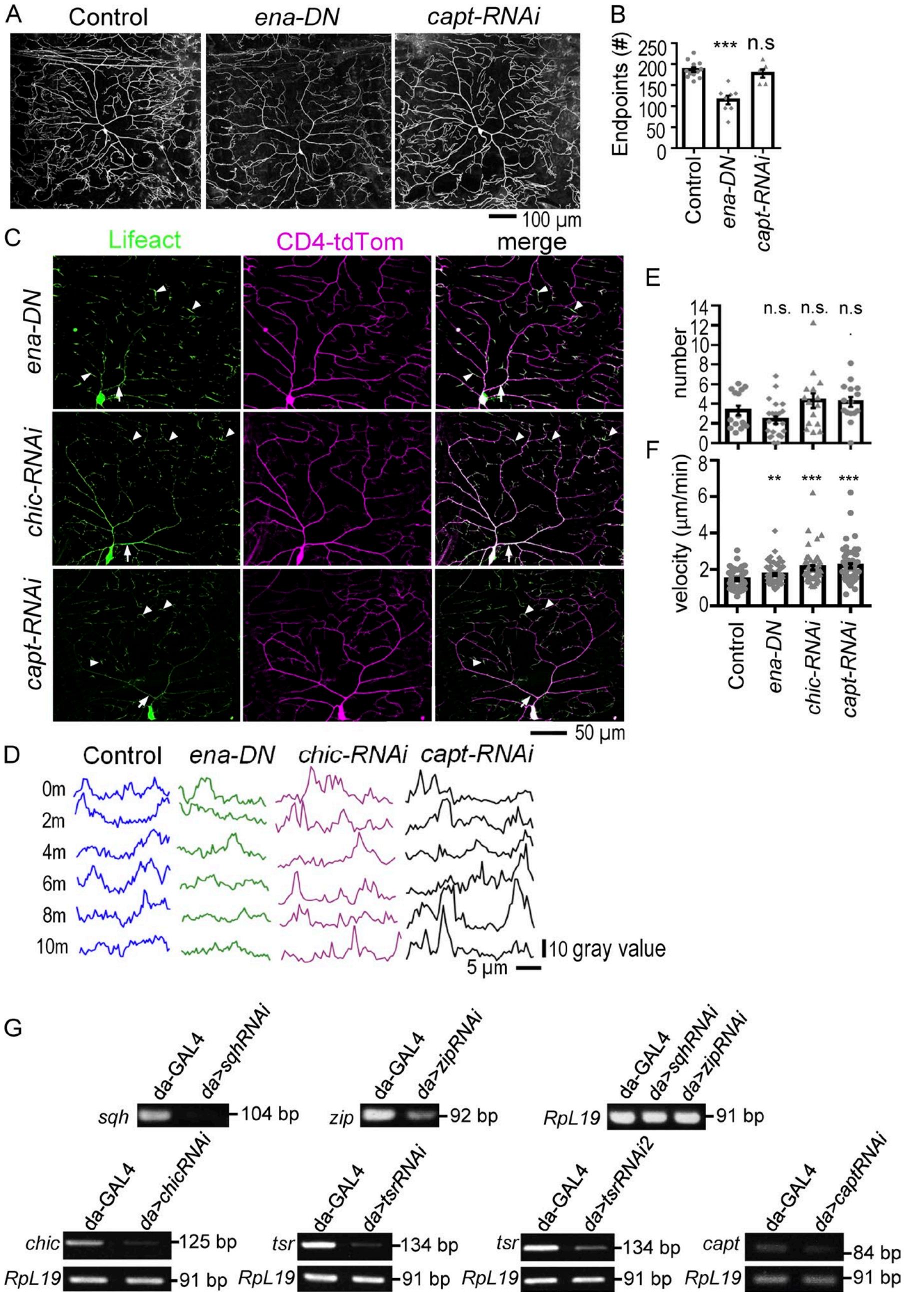

Figure S5. **Phenotypes in Ena dominant-negative, *chic-RNAi*, and *capt-RNAi* neurons. (A)** Images show dendritic trees in *ppk-GAL4* control, *ena-DN*, and *capt-RNAi* neurons. **(B)** Bar graph shows average numbers of dendritic endpoints present in the posterior dorsal region of the ddaC dendritic field. Numbers of neurons were 12 (control), 8 (*ena-DN*), and 6 (*capt-RNAi*). **(C)** Images show LifeAct distributions (green) in the CD4-tdTomato–labeled dendrites (magenta) of *ena-DN*, *chic-RNAi*, and *capt-RNAi* neurons. Merged images of two channels are at right. Arrows and arrowheads indicate higher LifeAct signals in proximal and terminal dendrites, respectively. **(D)** Line graphs show changes of LifeAct intensities in control (blue) and *ena-DN* (green), *chic-RNAi* (magenta), and *capt-RNAi* (black) dendrites over 10 min live imaging with 2-min intervals for each line. The intensities were assayed from linearized dendrites (x axis, µm; y axis, LifeAct gray value). Representative images were chosen from 15 dendrites of three neurons in one experiment (in addition to similar observations in Fig. 1 I) for control, 23 dendrites of five neurons in three experiments for *ena-DN*, and 15 dendrites of three neurons in two experiments for *chic-RNAi* and *capt-RNAi*. **(E and F)** Bar graphs show quantifications for actin blob numbers per 10 µm in 10 min (E) and velocities (F). In total, 52 actin blobs in 15 dendrites for control, 69 actin blobs in 23 dendrites for *ena-DN*, 50 actin blobs in 15 dendrites for *chic-RNAi*, and 62 actin blobs in 15 dendrites for *capt-RNAi* were scored. **(G)** Examining the knockdown efficiency of *sqh-RNAi*, *zip-RNAi*, *chic-RNAi*, *tsr-RNAi*, *tsr-RNAi2*, and *capt-RNAi*. *RpL19* is the loading control, and the predicted sizes (base pairs; bp) are indicated. Significance in comparison with control was determined by Student's *t* test. **, $P < 0.01$; ***, $P < 0.001$. Error bars represent SEM.

Table S1. **Screening for dendrite and actin blob regulators**

Description	Average ± SD (sample number)
Futsch intensity	Control; 1.7 ± 0.7 (*n* = 6)
	αTub84B-RNAi; 0.7 ± 0.3 (*n* = 7)
Anti-tubulin staining	Control; 1.1 ± 0.1 (*n* = 7)
	αTub84B-RNAi; 0.9 ± 0.1 (*n* = 8)
Jupiter-Cherry levels	Control; 2.2 ± 0.6 (*n* = 6)
	αTub84B-RNAi; 1.1 ± 0.2 (*n* = 6)
Tsr-GFP	72 h AEL control; 0.4 ± 0.2 (*n* = 9)
	72 h AEL *tsr-RNAi*; 0.2 ± 0.1 (*n* = 8)
	96 h AEL control; 0.2 ± 0.1 (*n* = 9)
Number in *αTub84B-RNAi* terminal dendrite (in 10 µm)	2.6 ± 1.6 (*n* = 25)
Velocity in *αTub84B-RNAi* terminal dendrite (µm/min)	1.5 ± 0.4 (*n* = 87)
Actin blob size in *αTub84B-RNAi* (µm)	3.2 ± 1.0 (*n* = 20)
Number in *UAS-Kat60* terminal dendrite (in 10 µm)	2.7 ± 2 (*n* = 20)
Velocity in *UAS-Kat60* terminal dendrite (µm/min)	1.9 ± 0.7 (*n* = 58)
Actin blob size in *UAS-Kat60* (µm)	3.2 ± 1.5 (*n* = 20)
Dendrites with LifeAct expression (per ddaC)	183 ± 19 (*n* = 9)
Dendrites with *GMA* expression (per ddaC)	175 ± 23 (*n* = 6)
ddac dendrites in *FRTG13* control (#)	141 ± 35 (*n* = 11)
ddac dendrites in tsr^{N121} (#)	70 ± 16 (*n* = 12)
ddac dendrites in tsr^{N96A} (#)	88 ± 28 (*n* = 12)
ddac dendrites in ena^{46} (#)	83 ± 12 (*n* = 15)
ddac dendrites in $shot^{3}$ (#)	22 ± 3 (*n* = 2)
ddac dendrites in *FRT40A* control (#)	157 ± 46 (*n* = 7)
ddac dendrites in $arpC1^{r337st}$ (#)	117 ± 12 (*n* = 8)
ddac dendrites in dia^{5} (#)	154 ± 16 (*n* = 5)
ddac dendrites in $capt^{E636}$(#)	161 ± 32 (*n* = 6)
ddac dendrites in *ppkGAL4* control (#)	199 ± 31 (*n* = 12)
ddac dendrites in *sqh-RNAi* (#)	191 ± 19 (*n* = 10)
ddac dendrites in *zip-RNAi* (#)	216 ± 15 (*n* = 10)
ddac dendrites in *ena-DN* (#)	115 ± 30 (*n* = 8)
ddac dendrites in *chic-RNAi* (#)	137 ± 24 (*n* = 9)
ddac dendrites in *capt-RNAi* (#)	178 ± 24 (*n* = 6)
Actin blobs in *ena-DN* terminal dendrite (in 10 µm)	2.3 ± 1.8 (*n* = 23)
Velocity in *ena-DN* terminal dendrite (in 10 µm)	1.7 ± 0.5 (*n* = 69)
Actin blob size in *ena-DN* (µm)	3.2 ± 1.4 (*n* = 20)
Actin blobs in *chic-RNAi* terminal dendrite (in 10 µm)	4.3 ± 2.9 (*n* = 15)
Velocity in *chic-RNAi* terminal dendrite (in 10 µm)	2.1 ± 0.9 (*n* = 50)
Actin blob size in *chic-RNAi* (µm)	2.5 ± 0.9 (*n* = 36)
Actin blobs in *capt-RNAi* terminal dendrite (in 10 µm)	4.2 ± 2 (*n* = 15)
Velocity in *capt-RNAi* terminal dendrite (in 10 µm)	2.2 ± 0.9 (*n* = 62)
Actin blob size in *capt-RNAi* (µm)	3.0 ± 1.1 (*n* = 27)

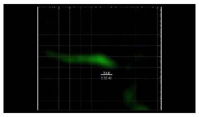

Video 1. **Actin blob propagation in the retrograde direction.** LifeAct is in green. Playback speed, 25 frames per second. Acquisition rate, 3 s per frame (Fig. 1 C).

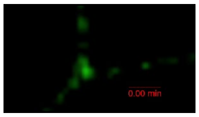

Video 2. **Anterograde movement of an actin blob.** LifeAct is in green. Playback speed, two frames per second. Acquisition rate, 18.7 s per frame. Bar, 2 µm (Fig. 1 D).

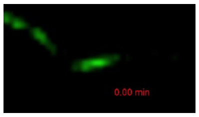

Video 3. **Passage of an actin blob through a branching site.** LifeAct is in green, and dendrite is in magenta. Playback speed, four frames per second. Acquisition rate, 18.8 s per frame. Bar, 2 µm (Fig. 1 E).

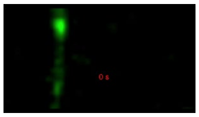

Video 4. **Actin blob splitting.** LifeAct is in green. Playback speed, two frames per second. Acquisition rate, 18.9 s per frame. Bar, 2 µm (Fig. 1 F).

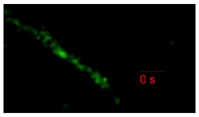

Video 5. **GMA propagating in the dendrite.** GMA is in green. Playback speed, three frames per second. Acquisition rate, 39.4 s per frame. Bar, 2 µm (Fig. S2 C).

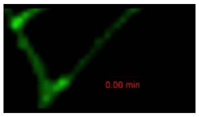

Video 6. **Anterogradely propagating actin blob seeds new dendrite branch.** Movement of an actin blob (green) in anterograde direction (0–2.48 min), stalling (4.97 min), and the emergence of a new branch (5.28 min). LifeAct is in green, and dendrite is in magenta. Playback speed, two frames per second. Acquisition rate, 18.7 s per frame. Bar, 2 µm (Fig. 2 A).

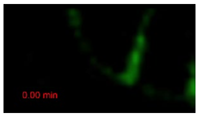

Video 7. **Retrogradely propagating actin blob seeds new dendrite branch.** Actin blob moves out of a retracting dendrite (0–1.55 min), stalls (2.8 min and 6.82 min), and a new dendrite emerges (7.45 min). LifeAct is in green, and dendrite is in magenta. Playback speed, two frames per second. Acquisition rate, 18.9 s per frame. Bar, 2 µm (Fig. 2 B).

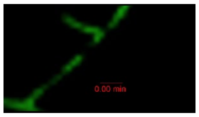

Video 8. **Two populations of actin blobs seed a new dendrite.** Two actin blobs merge at the stalling site (7.53 min) to induce dendrite emergence. LifeAct is in green, and dendrite is in magenta. Playback speed, two frames per second. Acquisition rate, 22.7 s per frame. Bar, 2 µm (Fig. 2 C).

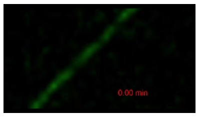

Video 9. **Local actin growth before dendrite branching.** F-actin accumulation (1.52–3.77 min) until a new dendrite emerges (4.15 min). LifeAct is in green, and dendrite is in magenta. Playback speed, two frames per second. Acquisition rate, 22.7 s per frame. Bar, 2 µm (Fig. 2 D).

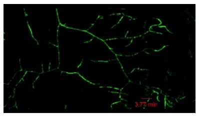

Video 10. **F-actin dynamics in dendrites of control, *G15S*, and *tsr-RNAi*.** LifeAct is in green. Playback speed: control, five frames per second (acquisition rate, 22.7 s per frame); *G15S*, eight frames per second (acquisition rate, 14.7 s per frame); *tsr-RNAi*, four frames per second (acquisition rate, 27.6 s per frame). Bar, 10 µm (Fig. 3 E).
